# Discovery and development of a safe and efficient COVID-19 mRNA vaccine, STP2104, using a novel capping library screening method

**DOI:** 10.3389/fimmu.2025.1571713

**Published:** 2025-06-09

**Authors:** Kanghyun Choi, Joo-Young Lee, Uk-Il Kim, So-Hyun Park, So-Hee Hong, Yoo-Jin Bang, Yun-Ho Hwang, Jong-Eun Lee, Suhyun Park, Soochong Kim, Sunhee Lee, Ye-Jin Yun, Tae-Gi Uhm, Inyoung Lee, Minju Kwon, EunJi Kwon, Suyeon Song, Yongkyoung Kweon, Heejene Kim, Eun-Young Oh, Jae-Yong Kim, Tae-Young Lee, Seo-Yeon Kim, Se-Eun Kim, You-Jin Kim, Seonock Lee, Ruijing Sun, Eun-Joo Lee, Gamin Kim, Hanah Lim, Byungkyun Kim, Jungho Kim, Dokeun Kim, Jae-Hwan Nam, Sang-Myeong Lee, Kyungjin Kim, Joo-Sung Yang

**Affiliations:** ^1^ R&D Center, ST Pharm Co., Ltd., Seoul, Republic of Korea; ^2^ College of Veterinary Medicine, Chungbuk National University, Cheongju-si, Republic of Korea; ^3^ Department of Microbiology, College of Medicine, Ewha Womens University, Seoul, Republic of Korea; ^4^ Department of Medical and Biological Sciences, The Catholic University of Korea, Bucheon-si, Republic of Korea; ^5^ Division of Infectious Disease Vaccine Research, Center for Vaccine Research, National Institute of Infectious Diseases, National Institute of Health, Korea Disease Control and Prevention Agency, Cheongju-si, Republic of Korea; ^6^ Department of Life Science, Sogang University, Seoul, Republic of Korea

**Keywords:** mRNA vaccine, COVID-19, SARS-CoV-2, STP2104, 5′-cap analogues, capping library screening, SmartCap ^®^

## Abstract

Messenger RNA (mRNA) vaccines represent a critical avenue for coronavirus disease 2019 (COVID-19) prevention. We developed a COVID-19 mRNA vaccine encoding a codon-optimized full-length ancestral spike (S) protein with a signal peptide, which employs our novel patented co-transcriptional 5′-capping reagent, SmartCap^®^. From the screening capping library of SmartCap^®^, an SC101 cap was selected to derive a novel mRNA vaccine, STP2104. An *in vitro* study of STP2104 incorporating SC101 revealed enhanced protein expression in both the cell lysate and culture medium, and an *in vivo* immunogenicity study revealed strong humoral and cell-mediated immune responses. STP2104 further displayed potent neutralizing activity in immunized mice as derived via the PRNT_50_ assay using the wild-type virus. We evaluated the protection efficacy of STP2104 using human ACE2 transgenic mice immunized and challenged with SARS-CoV-2 to acquire the survival rate, virus titration, and histopathology study data. These studies proved that STP2104 is potent enough to induce protective immunity. A novel capping library screening (CLS) method was successfully utilized for exploring the optimal 5′-cap reagent, which improves S gene expression with mRNA stability. The clinical phase 1 studies of STP2104 will prove its safety, tolerability, and immunogenicity as well as the safety of the novel 5′-cap analogue SC101 in humans.

## 1 Introduction

Coronavirus disease 2019 (COVID-19) is an infectious disease caused by severe acute respiratory syndrome coronavirus 2 (SARS-CoV-2). In 2019, a sudden outbreak of COVID-19 occurred, and it rapidly spread around the world. This disease’s symptoms include fever, coughing, fatigue, muscle and body aches, and headaches (1). From 2019 to 2022, almost 18 million people died as a result of COVID-19 (2). During this time, the World Health Organization (WHO) declared COVID-19 a pandemic, and it retained this status until 2022 (3). In 2023, the WHO declared COVID-19 endemic, marking the transition from the pandemic to the endemic phase (3, 4). The number of immunized people has increased through vaccination and natural infection, while mortality rates have decreased. However, the WHO has acknowledged that many countries, particularly in Africa, will require continued control to decrease infections (5, 6). Therefore, there is an increasingly urgent need to develop a prophylactic vaccine against SARS-CoV-2. During the pandemic phase, the Food and Drug Administration (FDA) approved mRNA vaccines, which were applied in a global vaccination campaign via Emergency Use Authorization (EUA) (7). The FDA-approved mRNA vaccines against SARS-CoV-2 were Comirnaty^®^ (BNT162b2 Pfizer-BioNTech vaccine) and SpikeVax^®^ (mRNA-1273 Moderna vaccine) (8). Moreover, vaccines from other manufacturers worldwide, including CVnCoV (CureVac) and PTX-COVID19-B (Providence Therapeutics), were also approved after clinical trials (9–11).

Most COVID-19 mRNA vaccines consisting of a 5′-cap, a 5′ untranslated region (UTR), a nucleoside-modified coding sequence (CDS) of the spike protein including a receptor-binding domain (RBD), a 3′ UTR, and a poly-A tail are encapsulated by lipid nanoparticles (LNPs) (12, 13). The early COVID-19 mRNA vaccines expressed ancestral strain full-length spike glycoproteins of SARS-CoV-2 (14). The 5′-capping structure of mRNA is essential for enhancing its stability, facilitating the transportation of mRNA, and protecting it from degradation (15). The protein expression level displayed by the same 5′-cap shows different patterns in different cell lines (16, 17). In addition, several different cap analogues drive protein expression, and this process is dependent on the cell lines, which have been evaluated for expression (18). For these reasons, the selection of a 5′-cap analogue for specific indications and a CDS is very critical in order to improve protein expression, which is important for developing mRNA vaccines and therapeutics.

To assist in developing a COVID-19 mRNA vaccine during the endemic phase, we applied our mRNA platform technology. We designed new 5′-cap structures and utilized these as a 5′-cap library. In this research, we evaluated our own patented co-transcriptional 5′-capping reagent, SmartCap^®^, which was selected by means of a capping library screening (CLS) method. Furthermore, we developed a COVID-19 mRNA vaccine candidate. Our mRNA vaccine encodes a codon-optimized full-length S-type spike protein with a signal peptide and uses SmartCap^®^ SC101 via the CLS method, constituting the first case of such an application worldwide. Herein, we compared the capping efficiency of several 5′-cap analogues, including SmartCap^®^, in cell lines to select the appropriate 5′-cap to be applied in an mRNA vaccine. Various SmartCap^®^s were compared by confirming the synthesis efficiency of three different mRNA payloads, and CLS was performed by comparing *in vitro* protein expression levels in various cell lines. The results derived from CLS are particularly reliable because they were based on comparison with the popular CleanCap^®^ reagent AG. To improve the delivery efficiency of the mRNA vaccine, we utilized an LNP system, which has been proven to perform well with an mRNA vaccine (19). We named our mRNA vaccine against the ancestral spike protein STP2104. We further analyzed the immunogenicity and safety of STP2104 using SmartCap^®^ at *in vitro* and *in vivo* levels. Our study proves that the STP2104 mRNA vaccine has potential as an mRNA-LNP vaccine against SARS-CoV-2.

Collectively, SmartCap^®^ can be a potent 5′-cap library platform for supporting the development of mRNA vaccines. In future, the pre-clinical toxicity of SmartCap^®^ SC101 in animals and its safety and efficacy in humans will be proven through GLP toxicity studies and clinical trials.

## 2 Materials and methods

### 2.1 Materials

The materials used included the following: STP2104 linearized plasmid DNA (Hanmi Pharmaceutical, Pyeongtaek, Republic of Korea); EGFP linearized plasmid DNA (ST Pharm, Ansan, Republic of Korea); 100 mM of adenosine 5′-triphosphate, cytidine 5′-triphosphate, guanosine 5′-triphosphate, and uridine 5′-triphosphate (Thermo Fisher Scientific, Waltham, MA, USA); SmartCap^®^ SC101 (2-amino-9-((2R,3R,4S,5R)-5-((((((((((2R,3R,4R,5R)-3-(((((2R,3S,4R,5R)-5-(2-amino-6-oxo-1,6-dihydro-9H-purin-9-yl)-3,4-dihydroxytetrahydrofuran-2-yl)methoxy)(hydroxy)phosphoryl)oxy)-5-(6-amino-9H-purin-9-yl)-4-fluorotetrahydrofuran-2-yl)methoxy)(hydroxy)phosphoryl)oxy)(hydroxy)phosphoryl) oxy)(hydroxy)phosphoryl)oxy)methyl)-3,4-dihydroxytetrahydrofuran-2-yl)-7-methyl-6-oxo-6,9-dihydro-1H-purin-7-ium) (ST Pharm, Ansan, Republic of Korea); CleanCap^®^ Reagent AG (TriLink Bio Thechnologies, San Diego, CA, USA); ARCA (TriLink Bio Thechnologies); T7 RNA polymerase (DYNEBIO, Seongnam, Republic of Korea); DNase I (TAKARA, San Jose, CA, USA); pyrophosphatase (Thermo Fisher Scientific); RNase inhibitor (TAKARA); 10X T7 RNA polymerase buffer (DYNE BIO); DNase I recombinant (TAKARA, Tokyo, Japan); and RNA Clean & ConcentratorTM-25 (Thermo Fisher Scientific). (6Z,16Z)-12-((Z)-dec-4-en-1-yl)docosa-6, 16-dien-11-yl 5-(dimethylamino)pentanoate (Lipid 10), di((Z)-non-2-en-1-yl) 9.9’-((2-(4-(9-(((Z)-non-2-en-1-yl)oxy)-9-oxononyl)piperazin-1-yl)ethyl)azanediyl)dinonanoate (STP1244), and PEG2000-c-DMA were chemically synthesized by ST Pharm in Republic of Korea. 1,2-distearoyl-sn-glycero-3-phosphocholine (DSPC) 1,2-Dioleoyl-sn-glycero-3-phosphoethanolamine (DOPE), cholesterol, and ethanol (99.5%) were purchased from Avanti polar lipid (USA) or Merck (Germany). Dulbecco’s modified eagle medium (DMEM), heat-inactivated fetal bovine serum (FBS), and penicillin–streptomycin were purchased from Hyclone (Wilmington, DE, USA). An EndoFree Plasmid Maxi Kit (QIAGEN cat.12362, Hilden, German), an AccuPrep PCR/Gel Purification kit (BIONEER, K-3037, Daejeon, Republic of Korea), and Amicon Ultra tubes (Merck Millipore, Cat. UFC210096, 100K.

### 2.2 Starting materials (plasmid DNA and linearized DNA) preparation

The plasmid DNA was prepared using an EndoFree Plasmid Maxi Kit (QIAGEN cat.12362). In brief, the transformant containing plasmid DNA was cultured in 200 mL of medium (1% seed culture with 2104, eGFP, hEPO, and Luciferase RCB stock) at 37°C for 16 h using kanamycin as a selection marker.

The linearized DNA was prepared using 5 µg of the template plasmid DNA, STP2104, eGFP, hEPO, or luciferase. The reaction solution was prepared as follows: 10X T buffer to 1X, 0.1% BSA, Sma1–1 U, NFW up to 100 µL, and incubation at 30°C for 2 h. The restricted sample was diluted to 100 ng/µL and loaded on 1% agarose gel with pDNA as a control to check for complete linearization. The linearized pDNA was purified using a PCR purification kit (AccuPrep PCR/Gel Purification kit, BIONEER, K-3037).

### 2.3 *In vitro* transcription reaction with various 5′-cap analogues: synthesis of SmartCap^®^ analogues

CleanCap^®^ reagent AG (N-7113) was purchased from TriLink. The reaction was composed of 5 mM each of an ATP solution, a CTP solution, and a GTP solution; 5 mM of an m1Ψ UTP solution; 1X of 10X T7 RNA polymerase solution; 4 mM of a 100 mM Cap solution; 5 µg of STP2104 eGFP, hEPO, or luciferase linearized plasmid DNA; 0.2 U of (stock 1 U/µL) YPP; 80 U of (stock 40 U/µL) RRI; and 4 KU of (stock 2 KU/µL) T7 RNA polymerase, and the volume was increased to 100 µL with NFW. The reaction was performed at 38°C ± 1°C for 4 h. Forty U of (stock 10 U/µL) DNase I was added to digest the plasmid DNA template and react for 1 h at 38°C ± 1°C. The 100 µL of the synthesized mRNA was dispensed into one Amicon Ultra tube (Merck Millipore, Cat. UFC210096, 100K). NFW was added to reach a total volume of 500 µL and mixed through pipetting. After discarding the solution under the filtered tube, NFW was pipetted in to reach 500 µL, mixed well, and centrifuged at 6000× *g* and 16°C for 10 min. The washing step was repeated until the A260/A230 value was 2.0 or higher. The filter was inserted upside down into the new tube, and we repeated the centrifuge step at 6000× *g* and 16°C for 2 min. The mRNA was transferred to a new e-tube. After measuring the concentration, mRNA was diluted to 200 ng and stored in a refrigerator at 75°C for 4 min and 4°C for 5 min before being loaded onto 1% agarose gel.

The SmartCap^®^ analogues were synthesized in 4 steps, as described below. In step 1, the dinucleotides were synthesized using protected nucleoside and phosphoramidite substituted with F or LNA at the 3′(R_3_) position of ribose. In step 2, the protected dinucleotide was synthesized via phosphorylation and oxidation. In step 3, we carried out deprotection reactions of the protected dinucleotide under acidic conditions for deprotecting the acetal group and then basic conditions for deprotecting the amide and cyanoethyl group to afford the deprotected dinucleotide. In step 4, the activated imidazole compounds (^m7^Gpp-Im, ^m7^G_3′-OMe_pp-Im, ^m7^G_2′-F_pp-Im, and ^m7^G_3′-F_pp-Im) and deprotected dinucleotide were synthesized under MgCl_2_ conditions and purified with DEAE Sephadex resin to obtain SmartCap^®^ analogues. The structures of the SmartCap^®^ analogues were identified via 400 MHz ^1^H NMR and 162 MHz ^31^P NMR spectroscopy (Bruker Avance III 400; Bruker Biospin, Rheinstetten, Germany) and liquid chromatography−mass spectrometry (Agilent 6120 LC/MS System; Agilent Technologies, Santa Clara, CA, USA). All synthesized SmartCap^®^ analogues were analyzed using high-performance liquid chromatography and found to be more than 95% pure.

### 2.4 Electrophoresis analysis

To check for linearization, the linearized DNA, with plasmid DNA as a control, was diluted to 100 ng and loaded on 1% agarose gel. The mRNA was diluted to 200 ng, denatured at 75°C for 4 min, and stored in a refrigerator for 5 min before being loaded onto 1% agarose gel.

### 2.5 Capping efficiency

5′-capping efficiency was analyzed using UPLC-DAD/Q-TOF equipment with an XBridge BEH C18 column (Waters, Milford, MA). In this process, a biotin-tagged probe that has a complementary sequence of mRNA binds to mRNA, and then RNase H cleaves the part of the biotin-tagged probe incorporated in mRNA, releasing the 5′-end.

### 2.6 Cell culture

HEK293T cells were maintained in Dulbecco′s modified Eagle′s medium (DMEM; Thermo Fisher Scientific) supplemented with 10% heat-inactivated fetal bovine serum (FBS; Sigma-Aldrich, St. Louis, MO, USA), GlutaMAX (Thermo Fisher Scientific) and 1% penicillin–streptomycin (Thermo Fisher Scientific) in a humidified 37°C incubator (Thermo Fisher Scientific) with 5% CO_2_. For LNP transfection, HEK293T cells (1 × 10^6^ cells/well) were plated in 6-well plates and cultured in Gibco™ Opti-MEM™ Reduced Serum Medium (Thermo Fisher Scientific) for 24 h.

Vero E6 cells were purchased from the American Type Culture Collection (ATCC), maintained in DMEM, and supplemented with 10% heat-inactivated FBS and 1% penicillin–streptomycin (Thermo Fisher Scientific).

### 2.7 Protein expression of SmartCap^®^-driven mRNA (eGFP, hEPO, and fLUC)

HEK293T and Huh7 cells were maintained in DMEM (Thermo Fisher Scientific) supplemented with 10% heat-inactivated FBS (Sigma-Aldrich), GlutaMAX (Thermo Fisher Scientific), and 1% penicillin–streptomycin in a humidified 37°C incubator (Thermo Fisher Scientific) with 5% CO_2_. For mRNA transfection, HEK293T cells (1 × 10^6^ cells/well) were plated in 6-well plates and cultured in Gibco™ Opti-MEM™ Reduced Serum Medium (Thermo Fisher Scientific) for 24 h. After 24 h of transfection, the fluorescence expressed by eGFP and hEPO in the cell cultures was analyzed by using an EVOS M5000 Microscope (Thermo Fisher Scientific) with a 10X scope and a Human Erythropoietin/EPO ELISA Kit (Bio-Techne^®^ R&D System, Quantikine DEPRU0, Minneapolis, MN, USA), respectively.

Luminescence was detected using a Bio-Glo Luciferase Assay Kit (Promega, Madison, WI, USA). For mRNA transfection, HEK293T and Huh7 cells (4 × 10^4^ cells/well) were plated in 96-well plates and cultured in Gibco™ Opti-MEM™ Reduced Serum Medium (Thermo Fisher Scientific) for 24 h. Bio-Glo™ reagent (Promega) was stored at room temperature for 4–6 h before use. A volume of Bio-Glo™ reagent equal to the cell volume (110 µL/well) was added to each assay well. After incubation at ambient temperature for 15 min, luminescence was measured using a microplate-reader.

### 2.8 LNP formulation

STP2104 was manufactured by mixing mRNA solution and lipid solution according to the previously reported papers (19–22) Briefly, STP2104 is a COVID-19 mRNA-LNP vaccine, which has SARS-CoV2 spike protein mRNA encapsulated by LNPs. The LNPs consist of ionizable lipids ((6Z,16Z)-12-((Z)-dec-4-en-1-yl)docosa-6, 16-dien-11-yl 5-(dimethylamino) pentanoate (Lipid 10) (ST Pharm, Republic of Korea), 1,2-distearoyl-sn-glycero-3-phosphocholine (DSPC, Avanti polar lipid, USA), cholesterol (Merck, Germany), and PEG_2000_-c-DMA (ST Pharm) in a molar ratio of 50:10:38.5:1.5). The lipid components dissolved in ethanol and the mRNA dissolved in acetate buffer (pH 5) were mixed in a 1:1 volume ratio using T-mixer. The obtained LNPs were diafiltered with PBS and ultrafiltered with a tris-based sucrose buffer using a tangential flow filtration (TFF) system. Finally, STP2104 bulk drug substance (DS) was sterilely filtered with 0.2 μm and fill-finished for drug product (DP) production.

In order to evaluate *in vivo* biodistribution in SmartCap^®^ screening, the lipid components consisting of an ionizable lipid (STP1244), DOPE, cholesterol, and C16 PEG2000-Ceramide were used in a composition ratio of 36.5:15:47:1.5 (mol%). This LNP formulation was prepared based on previous reports (23). The lipid components dissolved in ethanol and the mRNA dissolved in acetate buffer were mixed in a 1:1 volume ratio and at a flow rate of 12 mL/min using NanoAssemblr™ Ignite™ (Cytiva, Marlborough, MA, USA). The LNPs obtained were diafiltered with PBS, ultrafiltered with a tris/sucrose-based buffer at 3,500 rpm using an Amicon^®^ Ultra-15 Centrifugal Filter Unit (Merck Millipore, Darmstadt, Germany), and finally calibrated to the target concentration. The particle size of the manufactured LNPs was analyzed using a dynamic light-scattering device (Zetasizer Ultra, Malvern Panalytical, Mavern, UK). mRNA encapsulation efficiency and concentration were analyzed using a Ribogreen assay kit (ThermoFisher Scientific, Waltham, USA).

### 2.9 Western blot analysis after STP2104 *in vitro* transfection

STP2104 (mRNA 3 μg) was mixed with Opti-MEM up to a volume of 250 µL for 10 min at room temperature. This mixture was added dropwise into the culture medium and incubated for 24 h in an CO_2_ incubator. STP2104-transfected HEK293T cells were lysed at 24 h post-transfection in 150 µL of lysis buffer (50 mM Tris-HCl, pH 8.0, 2 mM EDTA, 1% NP-40, and 1x complete protease inhibitor cocktail solution (Roche Diagnostics)). Ten micrograms of cell lysate or the same volume of concentrates was loaded onto SDS-polyacrylamide gels. Gels were electro-transferred onto polyvinylidene difluoride (PVDF) membranes, treated with a blocking buffer (20 mM Tris-HCl pH 7.5, 137 mM NaCl, 0.1% Tween-20, 5% *w*/*v* non-fat dried milk) for 1 h at room temperature, and incubated with the primary antibody (anti-SARS-CoV-2 Spike RBD Polyclonal Antibody (2019-nCoV) (E-AB-V1006, Elabscience) or α/β-Tubulin Antibody (Cell Signaling Technology)) overnight at 4°C. After being washed three times with TBS-T buffer (20 mM Tris-HCl pH 7.5, 137 mM NaCl, and 0.1% Tween-20), membranes were incubated with the horseradish-peroxidase-conjugated secondary antibody (goat anti-rabbit IgG (ABclonal)) in blocking buffer for 1 h at room temperature. Detection was performed by means of chemiluminescence using Western Lightning Plus (PerkinElmer Life Sciences).

### 2.10 ELISA assay for secreted spike protein in culture media after STP2104 *in vitro* transfection

STP2104 (mRNA 3 μg) was mixed with Opti-MEM up to a volume of 250 μL for 10 min at room temperature. This mixture was inoculated into the culture medium and incubated for 24 h in a CO_2_ incubator. For the *in vitro* quantitative measurement of SARS-CoV-2 Spike S1 cleaved subunit protein concentrations in the cell culture supernatants, an ELISA assay was performed using the SARS-CoV-2 Spike S1 Protein ELISA kit (RK04154, ABclonal). After being washing (350 μL/well, a total of 3 times), the plates were incubated with serial dilutions of standard and sample solutions for 2 h at 37°C. After being washed three times, the plates were incubated with Working Biotin Conjugated Antibody solution for 1 h at 37°C. After standard washing steps, the plates were incubated with 100 μL of Working Streptavidin-HRP solution for 30 min at 37°C. After repeating the aspirations and washes, we prepared the plates using 100 μL of 3,5,3′5′-tetramethylbenzidine (TMB) as the substrate to detect antibody responses. The reaction was quenched using 50 mL of stop solution. As per the manufacturer’s instructions, the plates were read at 450, 570, and 630 nm on a microplate reader (Molecular Devices) to correct optical imperfections.

### 2.11 Quantitative RNA concentration using qRT-PCR

The STP2104 vaccine was manufactured in a batch of STP2104(2)-D-21002. This product was injected I.M. into 8–9-week-old male mice. Nucleic acids from each of 12 organs from 5 subjects were extracted at the time of vaccination (0 h) and 0.5 h, 6 h, 24 h, 48 h, 72 h, 120 h, 9 days, and 14 days after vaccination. The negative control group (Control) was a set of 3 unvaccinated mice, which were also sampled and analyzed. For the lymph nodes (LNs), five organs were pooled for nucleic acid extraction due to their small sizes and divided into two assay samples, and nucleic acids were extracted after homogenization. The amount of STP2104 mRNA in the extracted nucleic acids was quantified by means of quantitative reverse transcription PCR. The qRT-PCR reaction mixture was prepared using a Roche LightCycler 480 SYBR Green I Master kit and a specific primer set. The primers F and R amplified in the spike gene sequence of the STP2104 vaccine were designed and used as real-time PCR primers. The qRT-PCR reaction was conducted according to the LightCycler 480 SYBR Green I Master—Multi-well Plate 96 protocol. The amount of STP2104 mRNA (fg) per 1 ng of the extracted total RNA was calculated, and the distribution of the mRNA of each organ over time after vaccination was graphed. The limit of detection (LOD) concentration was 1 fg/µL.

### 2.12 *In vivo* biodistribution as determined via IVIS

*In vivo* distribution for SmartCap screening and STP2104 was approved by the Institutional Animal Care and Use Committee (IACUC) of KBIO Health (Approval no. KBIO-IACUC-2021–217 and KBIO-IACUC-2024-112). The firefly luciferase (fLUC) mRNA was I.M. injected at a concentration of 5 μg/50 μL into BALB/c mice. After the injection of the test substance, luminescence images (Bio-luminescence, BLI) were captured using IVIS Spectrum (PerkinElmer, USA) 10 min after we injected luciferin (15 mg/mL, 200 μL/mouse) intraperitoneally. Mice were anesthetized by making them inhale 1.5% isoflurane mixed with oxygen gas during photography, and the images were acquired in luminescence mode while the mice were in the ventral position. The body temperature of the mice was maintained using a warm plate attached to the equipment. Image analysis was conducted after all pictures had been taken (with this period lasting up to the 9th day) by adjusting to the corresponding luminescence intensity, and the ROI (region of interest) was set as the injection site. Total flux [p/s] at each time was obtained and compared.

### 2.13 Immunization of the animals

For *in vivo* immunogenicity, mouse studies were approved by the Institutional Animal Care and Use Committee (IACUC) of the Catholic University of Korea (Approval no. CUK-IACUC-2021-042). Female BALB/c mice were immunized twice at 4-week intervals with 1 μg, 5 μg, and 10 μg of the vaccine candidates. All the vaccine candidates were injected into the femoral muscle. To measure cellular-mediated and humoral immune responses, the spleen and blood were harvested 3 weeks after the last vaccination.

For cross-neutralizing antibody examination, we sought and gained approval for our mouse studies from the Institutional Animal Care and Use Committee (IACUC) of the Korea Disease Control and Prevention Agency (Approval no. KDCA-IACUC-21-025). Female BALB/c mice were immunized two times at 3-week intervals with 1 μg and 5 μg of the vaccine candidates. All vaccine candidates were injected into the femoral muscle. To evaluate cross-neutralizing antibody, blood was collected 2 weeks after the last vaccination, and serum was separated.

### 2.14 Fifty-percent plaque reduction neutralization titer 50

Neutralizing antibody (NAb) titration was conducted in a lab at the Korea National Institute of Infectious Diseases Good Clinical Laboratory Practice (KNIID GCLP-190). The method employed was adapted from a previously published paper and used with modifications (24). Briefly, Vero E6 (ATCC, catalogue #CRL-1586) was cultured in a 175T flask using 10% FBS + 1% Pen/Strep + DMEM. After removing the cell culture solution from the 175T flask, we washed the cells with 10 mL of DPBS (1×). The washing solution was removed and replaced with 5 mL of trypsin-EDTA. Cells were incubated for 2 min in a CO_2_ incubator. After incubation, 10 mL of 10% FBS DMEM was poured into the 175T flask, and the detached cells were transferred into a 50 mL tube. The cells were centrifuged at 1500 rpm for 3 min, and the supernatant was removed. The suspended cells were mixed in a 1:1 ratio using a 0.4% trypan blue dye. To analyze cell viability, we deposited 10 µL of a mixture of cells and trypan blue dye 0.4% (20 µL) onto a counting slide. The counted cells were suspended at 2 × 10^5^ cells/mL and aliquoted at 1 mL each into a 12-well plate. Serum inactivated at 56°C for 30 min was serially diluted 2-fold from the stock solution using 2% FBS + 1% Pen/Strep + DMEM. The wild-type virus (BetaCoV/Korea/KCDC03-NCCP43326/2020) and the Delta (NCCP 43389 SARS-CoV-2 B.1.617.1) and Omicron (NCCP 43411 SARS-CoV-2 BA.1.1) variants of SARS-CoV-2 were diluted to 4.5 × 10^2^ PFU/mL and mixed with serum in a 1:1 dilution ratio. The virus mixture containing the diluted serum was incubated in a CO_2_ incubator for 1 h. The serum-and-virus mixture was deposited into each prepared cell in a volume of 200 µL. The infected cells were incubated in a CO_2_ incubator for 1 h. After incubation, the mixed solution was removed, and 1 mL of the overlay medium (Overlay media: 4% FBS MEM (2X): 1.2% agar = 5: 5) was poured in. This plate was incubated for 3 days in the CO_2_ incubator. The overlay medium was removed, and crystal violet mixture (1 mL/well) was dispensed. The neutralizing antibody (NAb) titer was defined as the dilution factor corresponding to 50% plaque reduction compared to the positive control (virus only). The average number of plaques was counted for each dilution. The 50% neutralizing dose (ND_50_) titer was calculated using Karber’s formula, ND_50_ = 10^logND50^ (log_10_ND_50_ = m-Δ(∑p-0.5, where m denotes the highest dilution factor; Δ denotes log(dilution factor); and ∑p denotes the number of plaques/average plaque no. of the positive control (25). This experiment was performed in the BL3 laboratory (Facility no. KDCA-HP-21-3-02).

### 2.15 Captured Ab titration via ELISA

SARS-CoV-2 S1 and RBD proteins were added to the wells at 100 ng/50 μL in a 96-well plate and then incubated at 4°C for 12 h. The proteins were removed from the plate, and the 96-well plate was washed twice with washing buffer (PBS-T, PBS + 0.05% Tween20). Blocking buffer (PBS + 1% BSA buffer) was added at 100 μL/well and then incubated at 37°C for 2 h. Antigens diluted 2-fold from 1:320 (RBD) or 1:1280 (S1) were added at 100 μL/well and incubated at 37°C for 1 h. Goat anti-mouse IgG-horse radish peroxidase (HRP) antibody diluted to 1:5000 was added at 50 μL/well and then reacted at 37°C for 1 h. 1-Step™ Ultra TMB-ELISA solution (Thermo, 34028) was added to the incubation plate at 50 μL/well and reacted at room temperature for 10 min. The reaction was stopped by adding stop solution (GeneDEPOT, T3550-100) to the plate at 50 μL/well. Absorbance was measured at a wavelength of 450 nm using a microplate read, and the end-point titer was calculated.

### 2.16 ELISpot assay

The antigen-specific T-cell response has been suggested to be an important factor for increasing protective efficacy against virus infection. To evaluate the T-cell immune response induced by mRNA vaccination, an ELISpot assay was conducted using a SARS-CoV-2 (ancestral) peptide pool matrix. Each peptide was synthesized as a 15-mer with 9-mer overlapping with 1273 full-length amino acids of spike protein. All the peptides were pooled into 5 according to the order from the N-term. The peptide pool number 2 covers the receptor binding domain (RBD). An ELISpot assay was performed using an R&D systems EL485 kit. A 96-well plate coated with IFN-γ was blocked with cultured medium (200 μL/well) at room temperature for 20 min. Spleens harvested from the sacrificed mice were minced into single cells, and these cells were dispensed onto a plate containing 100 μL of the culture medium (5×10^5^ cells/well). The SARS-CoV-2 spike peptide pools and S1 peptide pool (Mabtech, Cat# 3629-1) were treated at 0.2 μg/peptide/well, and the stimulated cells were incubated in the CO_2_ incubator at 37°C for 18–20 h. After incubation, the plate was washed 4 times and treated with detecting antibody for 2 h. The plate was treated with enzyme-conjugated antibody at room temperature for 2 h. The addition of a 5-bromo-4-chloro-3-indolylphosphate/nitro blue tetrazolium (BCIP/NBT) substrate to the wells of microtiter plate and its incubation at room temperature for 1 h develops a colored product. Spots were counted using an Immunospot reader (Cellular Technology Limited., Shaker Heights, OH, USA).

### 2.17 SARS-CoV-2 *in vivo* challenge study in human ACE2 transgenic mice: qRT-PCR, plaque-forming assay

SARS-CoV-2 (NCCP43344) was obtained from the Korean National Culture Collection for Pathogens. SARS-CoV-2 was propagated and titrated using Vero E6 cells maintained in DMEM supplemented with 10% heat-inactivated FBS and 1% penicillin–streptomycin at 37°C in a 5% CO_2_ incubator. SARS-CoV-2 was handled in a biosafety level 3 (BSL3) facility at Chungbuk National University, and the corresponding procedure was approved by the Korean Centers for Disease Control and Prevention (KCDC-14-3-07). The challenge and survival study pertaining to STP2104 was approved by the Institutional Animal Care and Use Committee (IACUC) of Chungbuk National University (Approval no. CBNUA-2024-22-02).

Specific-pathogen-free transgenic (Tg) mice, namely, B6.Cg-Tg(K18-hACE2)2Prlmn/J mice, were obtained from Jackson Laboratories. The mice were randomly assigned to experimental groups. The hACE2 Tg mice were intramuscularly injected in the upper thigh with 1 μg, 5 μg, or 10 μg of STP2104 twice at 4-week intervals. Three weeks after the second immunization, the mice were inoculated with 5 × 10^4^ PFU/mouse via the intranasal route under isoflurane anesthesia. Body weight (BW), body temperature, and mortality were monitored daily for up to 13–14 days post-challenge (p.c.).

To assess viral burden and histopathology, the mice were euthanized at 4 days p.c., and samples were collected. Nasal washes were performed by flushing 20 μL of PBS through the nares, and the discharge was collected using the micropipette used for RNA extraction. The left lung lobes from the harvested whole lungs were immediately fixed in 10% neutral-buffered formalin solution for further histopathological examinations, and the right inferior lobes were homogenized for virus titration, while the right caudal lung lobes were used for RNA extraction.

Viral RNA was extracted from nasal washes and right-lung tissues using AccuPrep^®^ Viral RNA Extraction Kit (Bioneer, Daejeon, Republic of Korea) or RNAiso plus (Takara, Tokyo, Japan) according to the manufacturer’s instructions. qRT-PCR was performed as previously reported (11).

Plaque assays were performed to determine the SARS-CoV-2 infectious titers in the lung tissues. Briefly, the supernatant of homogenized tissues was subjected to 10-fold dilution from 10^−1^ to 10^−6^ and added into Vero-E6 cells grown in 12-well plates. After adsorption for 1 h, the virus inoculum was removed, and 1.5 mL of the overlay medium was added. After 2–3 days, the cells were fixed with 4% formaldehyde solution and stained with 0.4% crystal violet in 70% methanol in PBS. The viral titer was calculated after counting the number of plaques, and the limits of detection were as low as 25 PFU/mL.

The serum of the immunized mice was incubated with 100 PFU of the virus at 37°C for 1 h, and then the cells were inoculated with the virus–serum mixtures. After virus adsorption, an agar–overlay medium was added, and the plates were incubated at 37°C in a 5% CO_2_ incubator for 2 days. The cells were fixed with 4% formaldehyde and stained with 0.4% crystal violet, and the plaques were counted. The percentage of neutralization indicated the reduction value, which was calculated as the number of plaques in 100 PFU of the virus-infected wells per the number of plaques in the virus–serum-mixture-infected wells.

### 2.18 SARS-CoV-2 *in vivo* challenge study in human ACE2 transgenic mice: protection evaluation

Our challenge study was approved by the Institutional Animal Care and Use Committee (IACUC) of Chungbuk National University (Approval No. CBNUA-2024-22-02). Six-week-old female transgenic K18-hACE2 mice were purchased from The Jackson Laboratory (Bar Harbor, ME, USA). To examine the protective effect against viral infection, five groups (n = 10 per group) of K18-hACE2 mice were immunized with STP2104 twice in four-week intervals. After two weeks, the mice were anesthetized with a tiletamine/zolazepam and xylazine combination injected intraperitoneally. Subsequently, the mice were intranasally infected with about 100 LD_50_ (1 × 10^4^/plaque-forming units [PFU] in a volume of 30 µL) SARS-CoV-2. The infected mice were monitored daily for 14 days for weight loss and mortality. When their body weights had decreased to 75% of their initial body weights, the mice were anesthetized and humanely euthanized.

### 2.19 Histopathology and immunohistochemistry

Lung samples were fixed in 4% neutral phosphate-buffered formaldehyde (10% formalin) for 2 days and routinely processed and embedded in paraffin. Formalin-fixed paraffine-embedded blocks were sectioned at thicknesses of 5 μM with a microtome. Tissue sections were stained with hematoxylin and eosin (H&E). All slides were scanned using an Olympus VS200 Virtual Slide System (VS200; Olympus, Tokyo, Japan) and analyzed.

Pulmonary abnormalities were scored according to their representative microscopic lesions (**Supplementary Table S1**). The scoring criteria had a range of 0 to 3 according to the severity of each criterion: 0, none or minimal; 1, mild; 2, moderate; and 3, severe. The lung lesions were classified into 4 categories: (1) interstitial pneumonia; (2) perivascular lymphocytic infiltration; (3) vasculitis; and (4) peribronchiolar lesion. The final score of each criterion/category is shown as a heatmap and an average (**Supplementary Table S2**).

For immunohistochemistry, tissue sections were heated in a microwave for 5 min and sub-boiled at 90°C for 10 min in sodium citrate for antigen retrieval. Sections were then incubated overnight at 4°C with SARS-CoV-2 nucleocapsid protein (NP) antibody (Sino Biological, 40143-V-08B, PA, USA) in a 1:1000 dilution in antibody diluent. Biotinylated anti-rabbit IgG (Vectastain, PK-6101; Vector) was used to label SARS-CoV-2 NP. Tissue slides were incubated for visualization in 3,3′-diaminobenzidine (DAB, SK-4105, Vector Laboratories) at the concentration and time recommended by the manufacturers. Tissues were counterstained with methyl green. All slides were scanned using an Olympus VS200 Virtual Slide System (VS200; Olympus, Tokyo, Japan) and analyzed. The IHC scoring criteria have a range of 0 to 4 according to the area of the positive portion: 0, minimal or 0 to 10%; 1, mild or 10 to 25%; 2, moderate or 25 to 50%; 3, marked or 50 to 75%; and 4, severe or 75 to 100%.

### 2.20 Statistical analysis

Statistical analyses were performed via one-way ANOVA using GraphPad Prism10 (Version 10.4.0, GraphPad Software, Inc., Boston, MA. USA) and via *t*-tests using Microsoft Excel spreadsheet software (Microsoft Office 2013, Microsoft, Seattle, WA. USA).

## 3 Results

### 3.1 Selection of potent SmartCap^®^ via capping library screening

**Figure 1:**
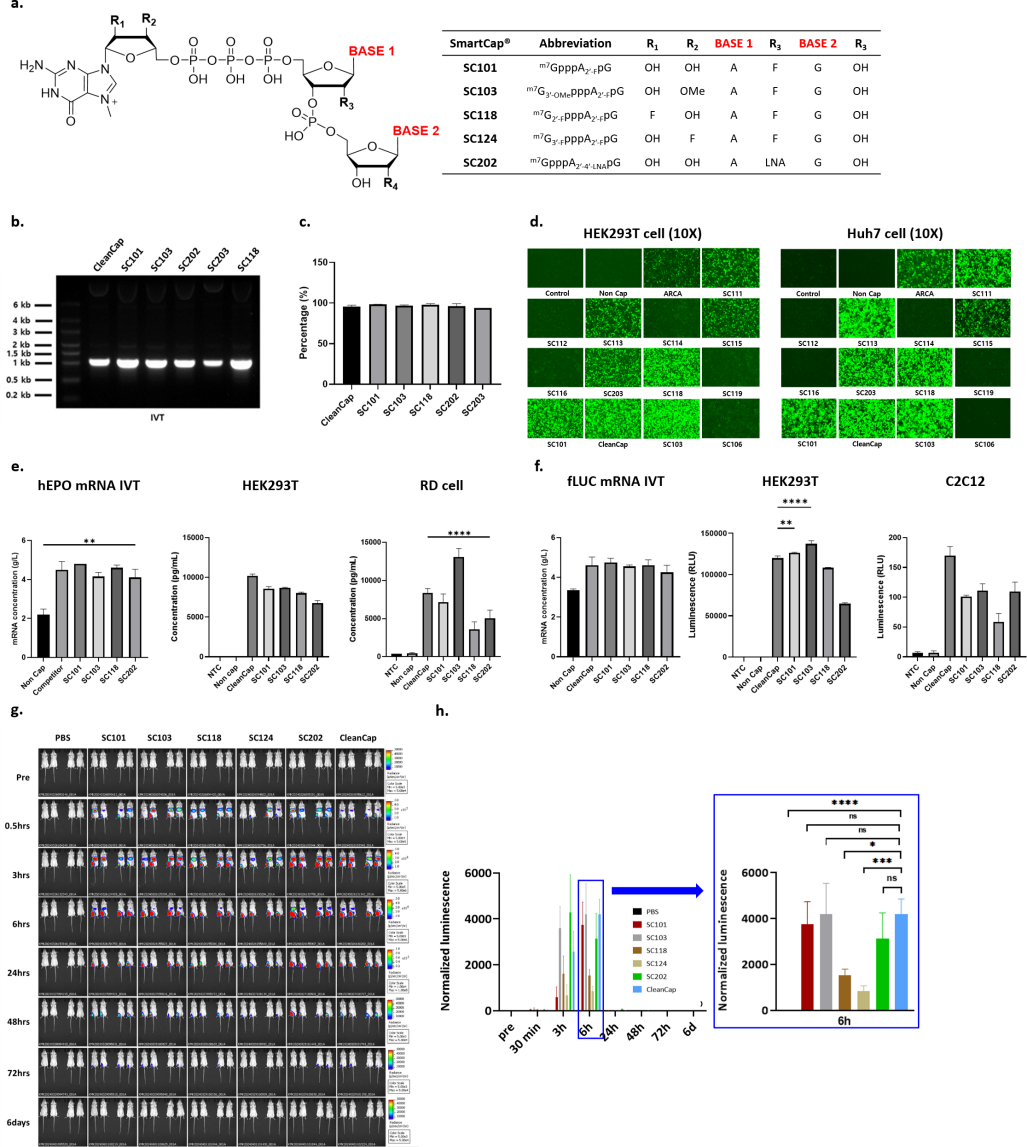
Evaluation of mRNA yield, capping efficiency, and potency of reporter genes using capping library screening. **(a)** SmartCap^®^ (SC) library composition with ribose and base modification. In synthesized eGFP mRNA, **(b)** IVT products on 1% agarose gel, **(c)** capping efficiency of several 5′-cap analogues, **(d)** naked eGFP mRNA transfection-mediated fluorescence in HEK293T and Huh7 were evaluated. **(e)** In hEPO mRNA synthesis, mRNA yield, and potency were determined via hEPO ELISA assay in HEK293T and RD cell culture media. **(f)** In fLUC mRNA synthesis, yield, and potency were determined via luciferase assay in HEK293T and C2C12 cell lysate. **(h)** Female BALB/c mice were intramuscularly injected with PBS or a 5 μg dose of fLUC (Capping with SmartCap^®^ library) mRNA LNPs. Luminescence was determined via IVIS (IVIS spectrum, PerkinElmer) up to 6 days p.i. Luminescence image from the whole body was measured over time for up to 6 days p.i. **(h)** Total flux of the whole body was plotted over time. Statistical analyses were performed using one-way ANOVA (*p < 0.05, **p < 0.01, ***p < 0.001, ****p < 0.0001, FF ns: no significant).

We invented an mRNA platform technology involving novel 5′-cap analogues based on ST Pharm’s strong nucleic chemistry background and ample experience in the monomer and oligonucleotide CDMO industry. We improved capping and transcriptional efficiency, thus preventing mRNA degradation and minimizing innate immune response induction (26). The capping library consists of over 30 different novel 5′-cap analogues. On the basis of an m^7^ guanosine triphosphate backbone, the 2′ position of the 5′-cap ribose or the second and third ribose was modified. For base modification at the second and third ribose, several different bases were introduced (**Figure 1a**). Our novel 5′-cap analogue was registered and named SmartCap^®^ (SC).

We evaluated the SC library for the mRNA yield and potency of SC with three reporter mRNAs in two human cell lines. In eGFP mRNA synthesis, the mRNA yield obtained using the analogues SC101, SC103, and SC118 of the capping library was higher and, in some cases, comparable to that for CleanCap^®^ (CC), except with respect to SC203 (**Figure 1b**). The 5′-capping efficiency of the synthesized eGFP RNA was over 94%, which is comparable to that for CC (**Figure 1c**). eGFP expression was compared by means of fluorescent microscopy in HEK-293T and Huh7 cells at 24 h p.t. The fluorescence levels of SC101, SC103, SC118, and CC were comparable, and we observed higher fluorescence than that in the other SCs in the HEK293T cell line. Interestingly, SC111- and SC113-mediated eGFP expression was higher in Huh7 cells than in HEK293T cells (**Figure 1d**). Furthermore, we observed hEPO expression levels from selected 5′-cap analogues in HEK293T and RD cell lines. In hEPO mRNA synthesis, the mRNA yield was comparable and over 4 g/L among the tested 5′-cap analogues. In HEK293T cells, the secreted hEPO in cell culture media was measured by means of ELISA, showing that CC-driven hEPO expression was higher than that driven by SCs. However, SC103-mediated hEPO secretion was higher than that mediated by other cap analogues in the RD cells (**Figure 1e**). For fLUC reporter mRNA, the yield was over 4 g/L. Luciferase activity was measured with cell lysate from the HEK293T and C2C12 cell lines. In the HEK293T cells, SC101- and SC103-mediated fLUC expression was higher than that mediated by CC. Although fLUC expression levels were lower in mouse C3H muscle myoblast-derived C2C12 cells, CC induced the highest fLUC activity (**Figure 1f**). C2C12 cells showed different protein expression tendencies for the selected caps. The *in vitro* protein expression levels among the selected cap analogues were compared using one-way ANOVA statistical analysis.

We conducted *in vivo* biodistribution analysis after administering intramuscular (I.M.) injections of fLUC mRNA LNPs for SC library screening (**Figures 1g, h**). PBS was used as a negative control, and naked, non-capped fLUC mRNA and naked SC101 fLUC mRNA, which were not formulated with LNPs, were injected. According to the time course after injection, the total flux level was measured. No expression was observed in the negative control group. In the case of the fLUC mRNA LNPs using various SCs, compared to fLUC mRNA capped with CC, the SC202 fLUC mRNA LNPs showed approximately 1.8-fold higher expression as determined via AUC ((photons/second) × hours). The total flux level was shown to be in the order of SC202 > SC103 > CC and SC101 (**Figure 1h**). Although other SCs led to higher levels of expression, SC101 demonstrated an expression level comparable to that of CC.

Based on the capping library screening, we decided to use SC101 as a 5′-cap analogue for further COVID-19 prophylactic mRNA vaccine development.

### 3.2 Construction of a COVID-19 mRNA prophylactic vaccine, STP2104, and potency

**Figure 2:**
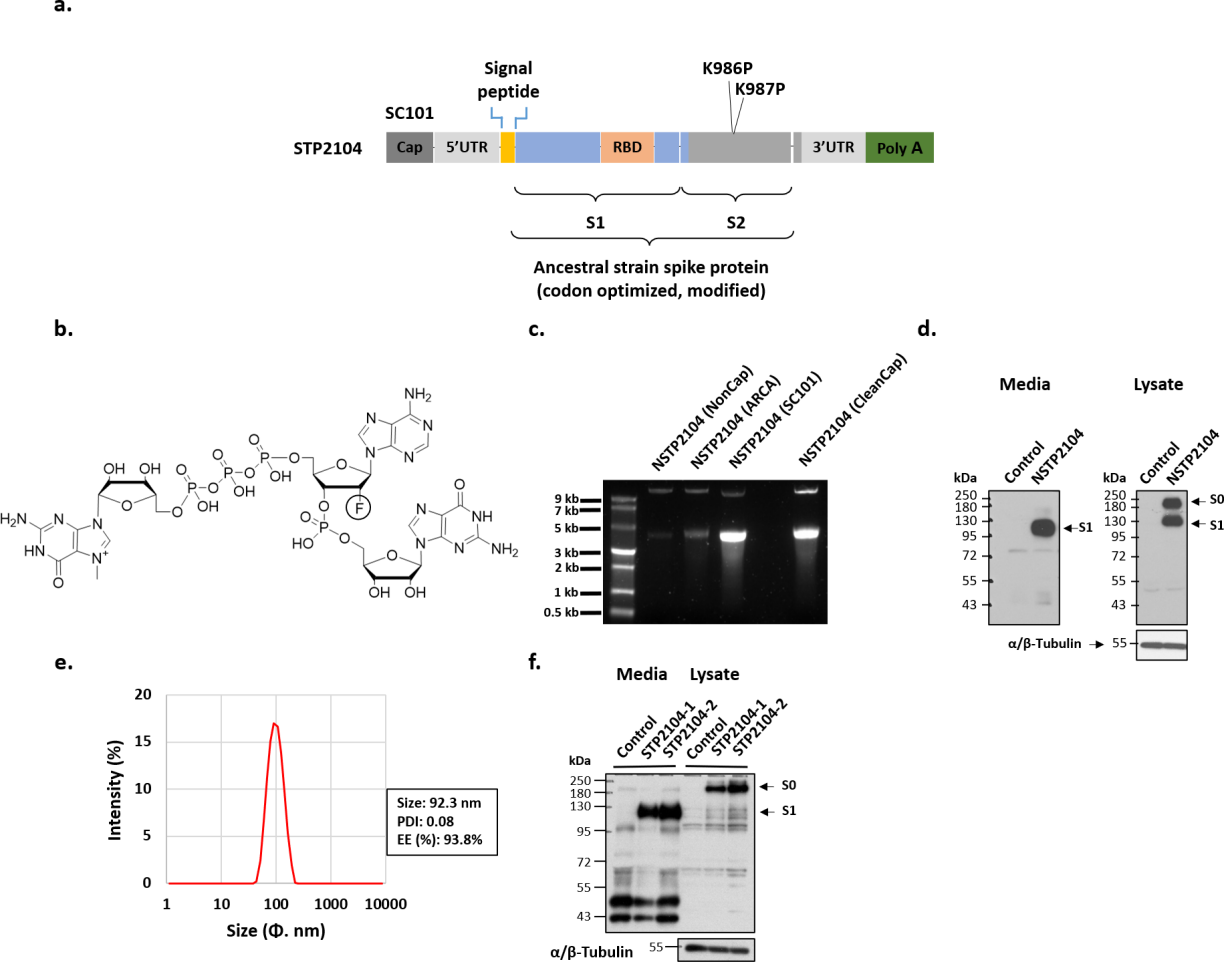
SARS-CoV-2 spike protein mRNA construction and protein expression of NSTP2104 (naked mRNA) and STP2104 (mRNA-LNPs). **(a)** Schematic diagram of STP2104 mRNA’s molecular structure. **(b)** Chemical structure of SC101. **(c)** Visualization of synthesized spike mRNA obtained using ARCA, SC101, and CleanCap. **(d, f)** Spike protein expression in HEK293T cells after transient transfection with NSTP2104 **(d)** or treatment of STP2104 mRNA LNP **(f)**. **(e)** Physicochemical properties of STP2104 (size, PDI, and encapsulation efficiency (EE)).

We developed a COVID-19 mRNA vaccine, STP2104, as a basic vaccine. The schematic diagram included in this Section depicts the molecular structure of the STP2104 mRNA comprising the SC101 binding site, both the 5′ and 3′ UTRs, the signal peptide, the coding sequence, and the poly-A tail (**Figure 2a**). The coding sequence is the full length of the ancestral strain spike, and it is fused to the signal peptide, allowing higher protein expression and secretion, as previously noted (27).

SC101 is a co-transcriptional capping analogue for the *in vitro* transcription of 5′-capped mRNA. It is composed of trinucleotides. The first nucleotide is N-7-methylguanosine, connected to the second nucleotide through a 5′-5′-triphosphate bridge. The second nucleotide features a fluorine substitution at the 2′ position of the ribose. Adenine and guanine are incorporated at the base of the second and third ribose, respectively (**Figure 2b**).

An ancestral SARS-CoV-2 spike naked mRNA (NSTP2104) was synthesized via an *in vitro* transcription (IVT) reaction using SC101, CleanCap, and an anti-reverse cap analogue (ARCA). SC101 synthesized the spike mRNA NSTP2104 more efficiently than ARCA and equally as efficiently as CleanCap (**Figure 2c**). Spike protein expression was analyzed with naked mRNA (NSTP2104) and LNP-formulated mRNA (STP2104). The NSTP2104 was transiently transfected into HEK293T cells, and an expression of 190 kDa for the full-length spike protein (S0) and 110 kDa for cleaved subunit 1 (S1) was confirmed by means of Western blot analysis using an RBD-specific polyclonal antibody. Precursor S0 and cleaved S1 spike proteins were identified in the cell lysate, and only the S1 fragment was detected in the media (**Figure 2d**). The formulated NSTP2104 with LNPs presented with physicochemical properties such as a size of less than 100 nm, a PDI of less than 0.1, and an encapsulation efficiency (EE) of over 90% (**Figure 2e**). The potency of STP2104 was evaluated with two different lots, and it was found that most of the cleaved S1 protein was detected in the culture media (**Figure 2f**). In addition, the secreted SARS-CoV-2 S1 protein concentration (6.32 ± 0.09 ng/mL) was quantified by means of ELISA analysis.

### 3.3 *In vivo* biodistribution of Residual mRNA after I.M. injection of STP2104

**Figure 3:**
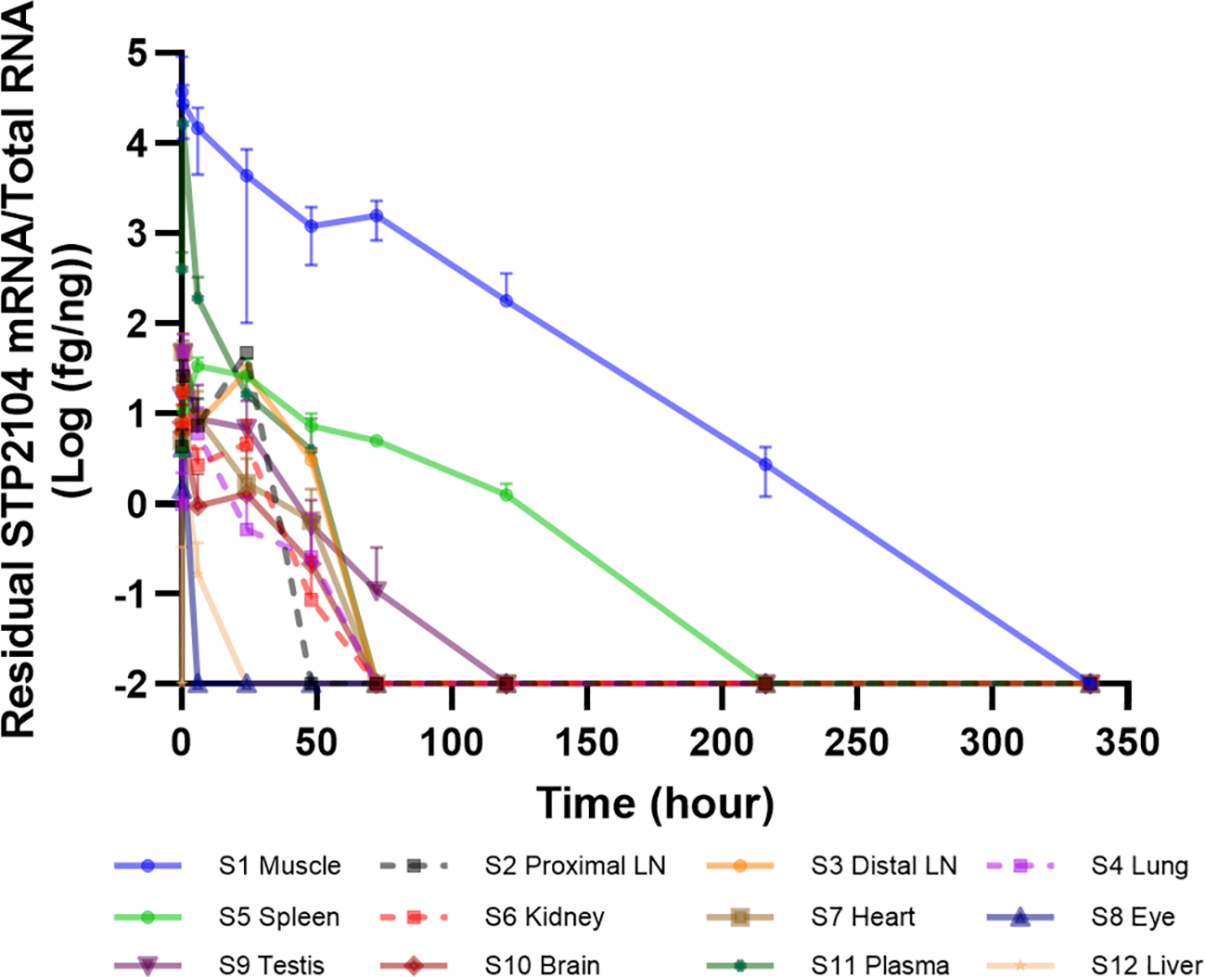
*In vivo* STP2104 mRNA biodistribution determined via qRT–PCR. Residual NSTP2104 (naked spike mRNA) was analyzed in multiple tissues/organs for up to 14 days after the I.M. injection of STP2104 (mRNA–LNP). Total RNA from each sample was extracted, and synthesized cDNA was analyzed to determine NSTP2104 mRNA levels via qRT–PCR. LN, lymph node.

We evaluated mRNA biodistribution after the I.M. injection of STP2104. The amount of mRNA was quantitated via qRT-PCR with a known concentration of an RNA standard. The total RNA from twelve tissues/organs was extracted according to the time course. The detection level was highest at the injection site (muscle) and second highest in the plasma, and less than 50 fg/ng of mRNA was detected in the remaining tissues/organs in the early period after injection. Five days after injection, no mRNA was detected in the rest of the tissues/organs except for the muscle and spleen. The mRNA copy number was no longer detected in the spleen and muscle 10 and 14 days after injection, respectively (**Figure 3**).

### 3.4 *In vivo* biodistribution of luminescence after I.M. injection of fLUC mRNA LNPs

**Figure 4:**
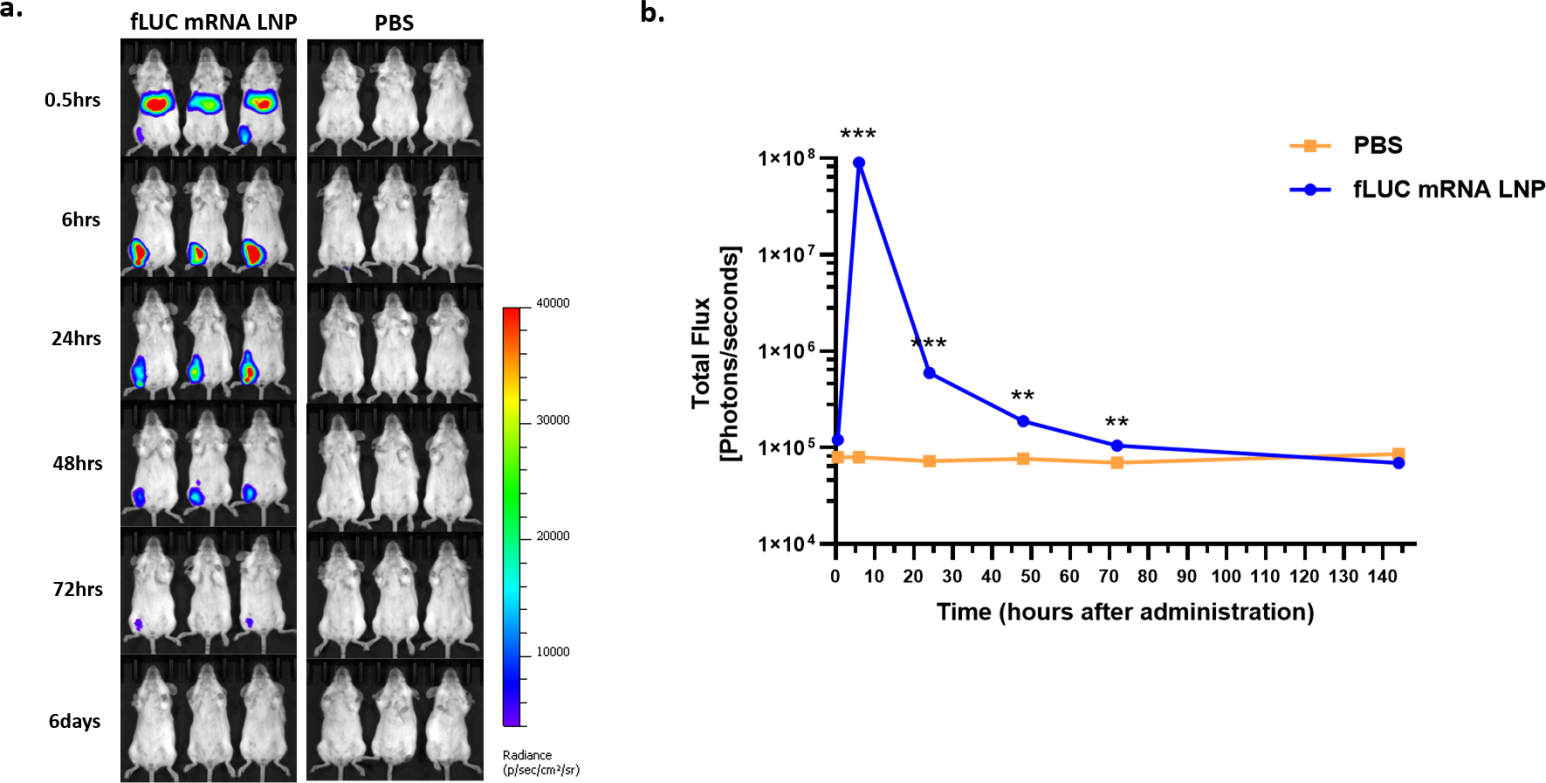
*In vivo* biodistribution of firefly luciferase (fLUC) mRNA LNPs determined via IVIS. Female BALB/c mice were intramuscularly injected with PBS or a 5 μg dose of STP2104. Luminescence was determined via IVIS (IVIS spectrum, PerkinElmer) for up to 6 days post-injection (p.i.). **(a)** Luminescence image from whole body over the course of up to 6 days p.i. **(b)** Total flux of the whole body over time. Each total flux in ROI and the results were statistically analyzed by means of a *t*-test. (**p < 0.01, ***p < 0.001).

The *in vivo* biodistribution of LNP-formulated luciferase reporter mRNA was tested by means of the I.M. injection of fLUC mRNA LNPs or PBS as a negative control. *In vivo* luminescence imaging was analyzed over time, and the changes in luminescence intensity in the whole body were evaluated (**Figure 4a**). In the fLUC-mRNA-LNP-injected mice, luminescence in the liver peaked at 30 min after injection and disappeared at 6 h post-injection (p.i.). At the injection site, luminescence peaked at 6 h p.i. and was detected until 72 h p.i. (**Figures 4a, b**).

### 3.5 STP2104 vaccination induced potent humoral immune responses as determined by captured IgG and by neutralizing Ab titers

**Figure 5:**
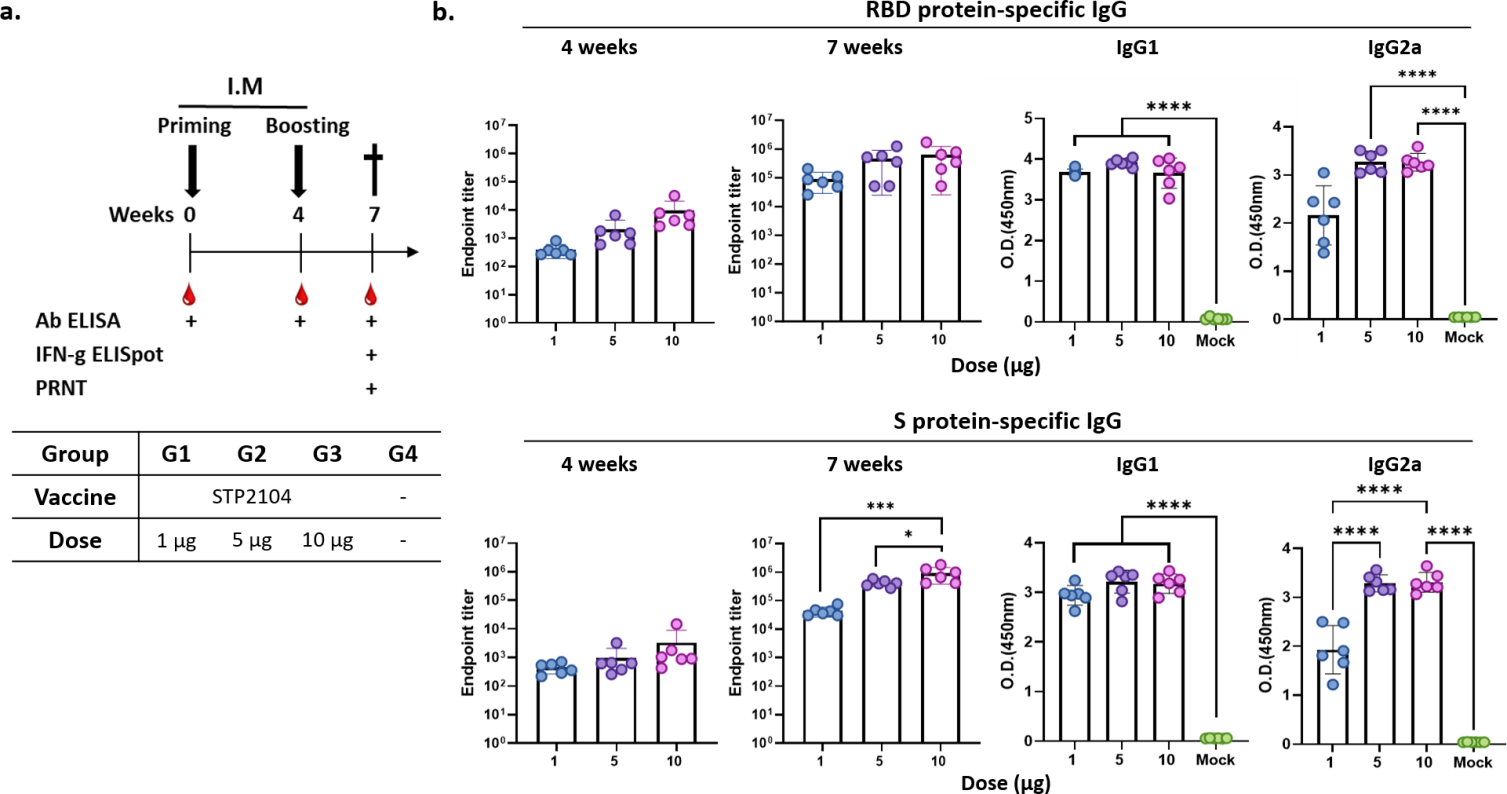
Evaluation of humoral immune response after immunization with STP2104. **(a)** Overall study scheme. Female BALB/c mice were intramuscularly immunized with 1 μg, 5 μg, or 10 μg twice in 4-week intervals. (n = 6/group) **(b)** Captured antibody titers for both the RBD and spike protein were measured twice 4 weeks after priming and 3 weeks after boosting. Statistical analysis was performed using one-way ANOVA (*p < 0.05, ***p < 0.001, ****p < 0.0001).

The mice were I.M. immunized twice with 1 μg, 5 μg, or 10 μg of STP2104 in 4-week intervals. The obtained antibody titers for both RBD and spike recombinant protein were measured 4 weeks after priming and 3 weeks after boosting based on the immunization scheme (**Figure 5a**). There was no statistically obvious dose-dependent endpoint titer increase in the RBD recombinant protein-specific total IgG level at 4 weeks and 7 weeks. Priming/boosting immunization exhibited a higher IgG titer than priming in S protein-specific IgG ELISA. The spike-protein-specific total IgG titer increased dose- and frequency-dependently (**Figure 5b**). The average RBD- and S-specific IgG endpoint titers from the 10 µg dose group were 10^3.5^~10^4^ at 4 weeks after priming and about 10^6^ at 3 weeks after boosting (**Figure 5b**). Therefore, STP2104 immunization induced a strong humoral immune response. In addition, IgG isotyping was assayed, and IgG2a/IgG1 was determined to be close to 1 in S-protein-coated Ab ELISA.

**Figure 6:**
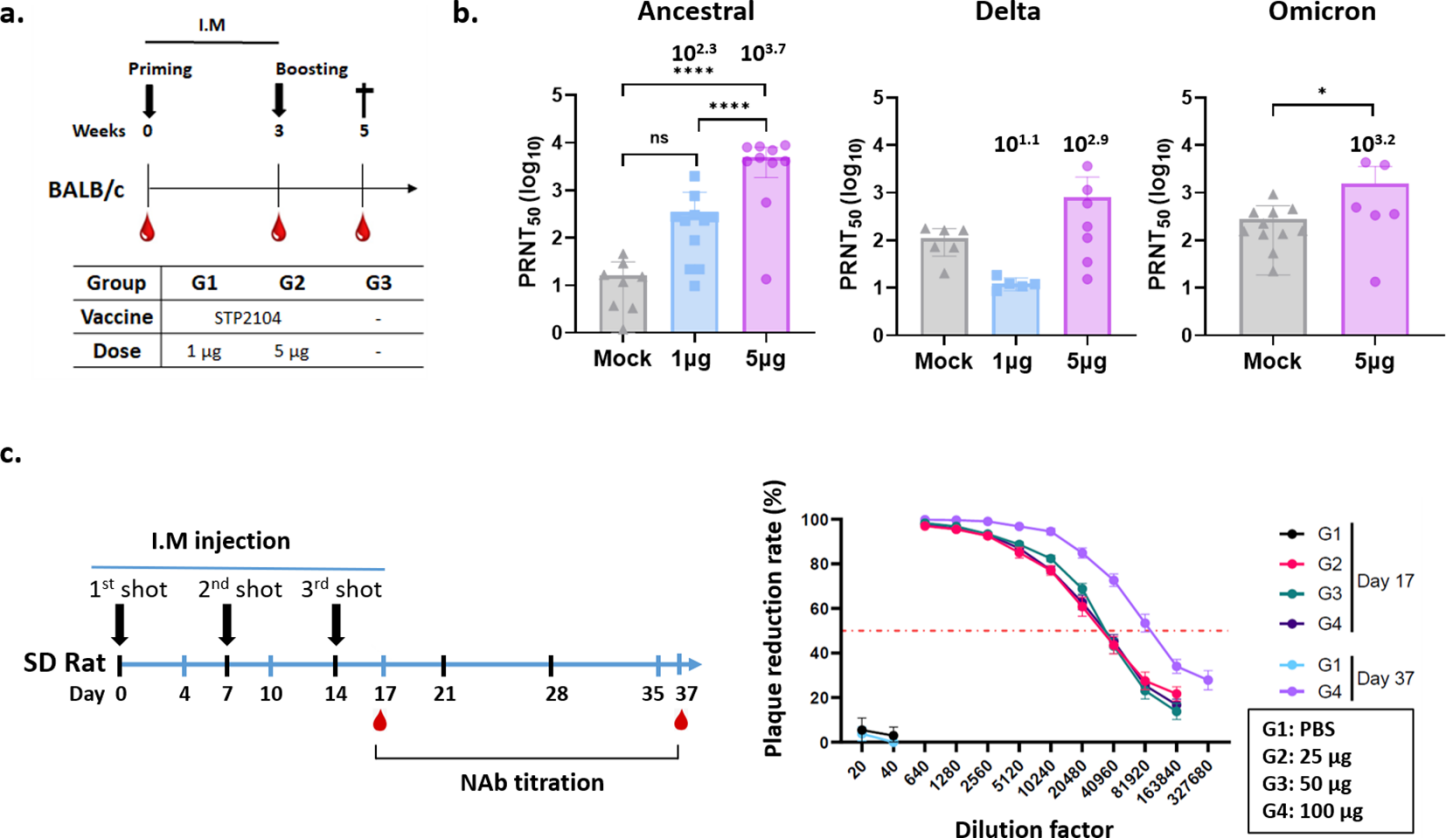
Evaluation of neutralizing antibody titers after immunization of BALB/c mice and SD rats with STP2104. **(a)** Female BALB/c mice were intramuscularly immunized with 1, 5, or 10 μg of STP2104 at 3-week intervals (n = 6/group). Serum samples taken 2 weeks after boosting were measured in terms of **(b)** NAb titers against ancestral, delta, and omicron variants through PRNT analysis. **(c)** SD rats were injected three times with 25, 50, or 100 μg of STP2104 at 1-week intervals. Neutralizing Ab titers were measured in serum samples taken from three different dose groups and PBS on day 17 after three intramuscular injections and only from the 100 μg dose group on day 37 after a 3-week recovery period. *p < 0.05, *****p < 0.0001, ns means “no significant”.

Two weeks after the second dose, serum analysis revealed a dose-dependent, significant increase in neutralizing antibody titers against the ancestral virus induced by STP2104, with a mean PRNT50 value of 10^3.7^, indicating a high level of antibody response. To assess cross-neutralizing activity against other viral strains, PRNT50 assays were performed for the Delta and Omicron variants using the same methodology. The results showed no detectable neutralizing antibodies against the Delta variant. In contrast, for the Omicron variant, the mean PRNT50 at the 5µg dose was 10^3.2^ (**Figures 6a, b**).

To confirm the immunogenicity of STP2104 in other animals, namely, the *Rattus* genera other than Mus, Sprague Dawley^®^ *(*SD) rat sera harvested from the GLP 3-week repeated toxicity study for STP2104 were analyzed for neutralization activity (**Figure 6c**). The sera were harvested on day 17 after three I.M. injections and on day 37 after a 3-week recovery period and analyzed for PRNT_50_. The NAb titer increased with time. On day 17 after three injections, the PRNT_50_ was 10^4.3^ for all three dose groups, namely, 25, 50, and 100 µg. After a 3-week recovery period, the PRNT_50_ was 10^4.9^ for the 100 µg injection group (**Figure 6c**).

### 3.6 STP2104 vaccination induced an Ag-specific cell-mediated immune response

**Figure 7:**
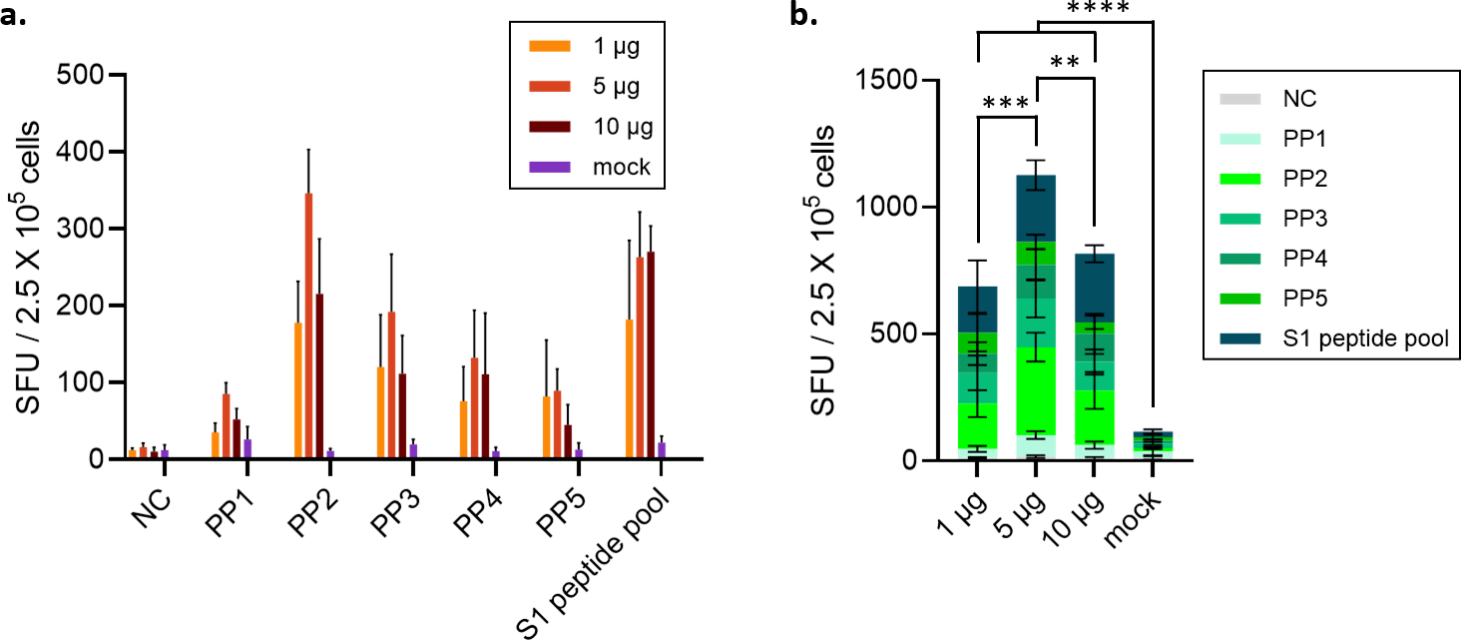
Evaluation of IFN-γ-secreting SFU after immunization of animals with STP2104. **(a)** Female BALB/c mice were intramuscularly immunized with 1, 5, or 10 μg at 4-week intervals. (n = 6/group). **(b)** Four weeks after the second vaccination, the number of T cells secreting IFN-γ was measured using the ELISPOT assay. Statistical analysis was performed using one-way ANOVA (**p < 0.01, ***p < 0.001, ****p < 0.0001).

The spike-specific cellular immune response was evaluated by means of the IFN-γ ELISpot assay with the stimulation of spike peptide pools (PPs) as a recall antigen, encompassing the full-length spike protein (1273 amino acids). Five peptide pools stimulated IFN-γ secretion from splenocytes isolated from all the vaccinated BALB/c mice groups. The highest spot-forming unit (SFU) value for IFN-γ-producing T cells was observed in the 5 µg dose group stimulated with peptide pool #2 covering the RBD region (**Figures 7a, b**). In the 10 µg-immunized group, the SFU value was lower than that of the 5 μg dose group (**Figure 7b**).

### 3.7 STP2104 vaccination induced CD4^+^/CD8^+^ Memory T-cell responses

**Figure 8:**
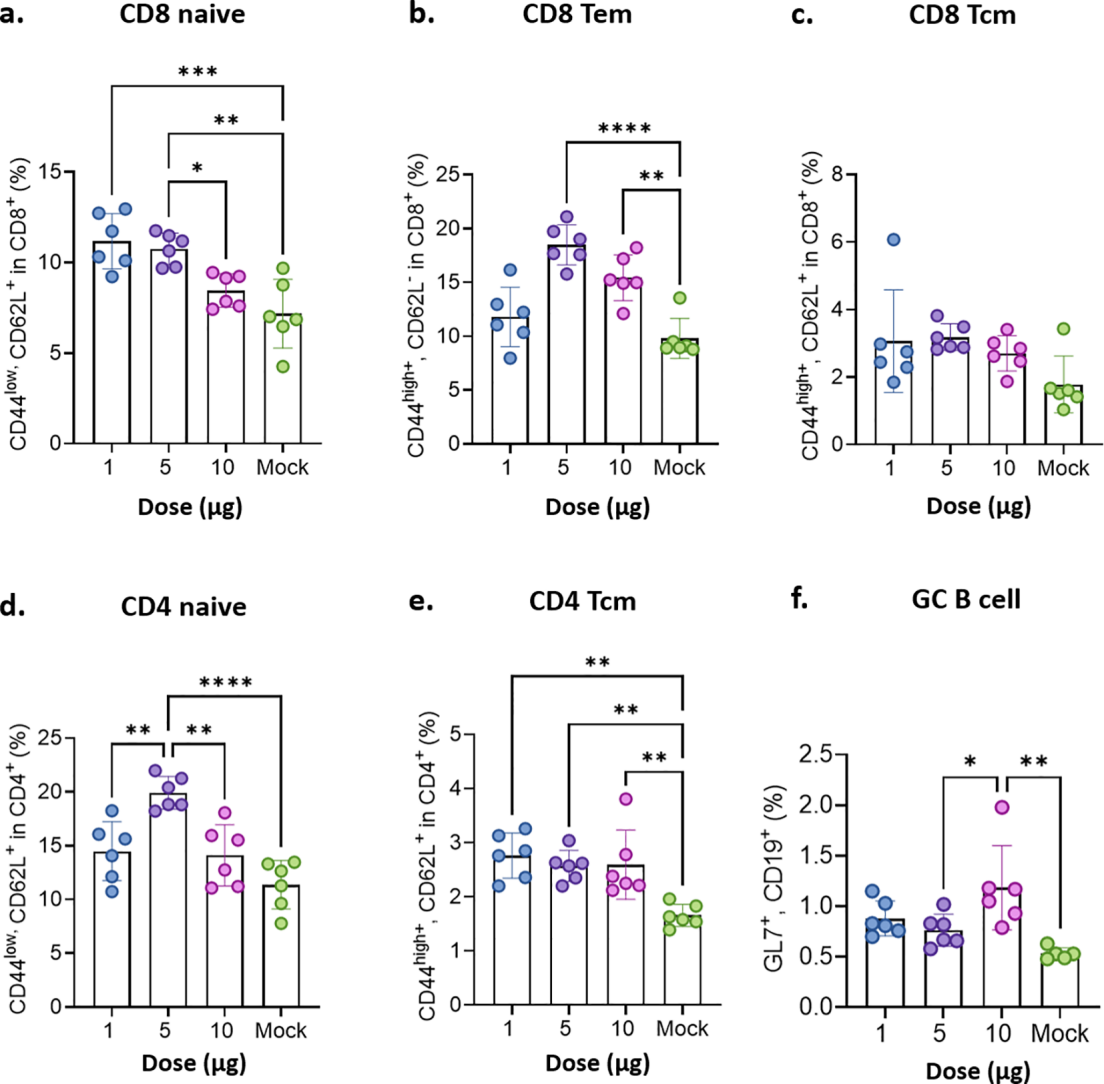
Evaluation of memory T cells’ immune response after the mice were immunized with STP2104. Female BALB/c mice were intramuscularly immunized twice with a 1 μg, 5 μg, or 10 μg dose of STP2104 at 4-week intervals. (n = 6/group). At 3 weeks after the second immunization, CD4/8 T cell subpopulations including **(a)** CD8 naïve, **(b)** CD8 Tem, **(c)** CD8 Tcm, **(d)** CD4 naïve, **(e)** CD4 Tcm, and **(f)** GC B cells were analyzed by means of flow cytometry after stimulation with spike protein peptide pool number 2. (Tem: effector memory T, Tcm: central memory T, and GC: germinal center). Statistical analyses were performed using one-way ANOVA. (*p < 0.05, **p < 0.01, ***p < 0.001, ****p < 0.0001).

STP2104 mRNA vaccination increased the percentage of both naïve CD44^low^/CD62L^+^ CD4 and CD8 cells at a 5 µg dose (**Figures 8a, d**). Increased percentages of CD4 central memory T cells (Tcm) were observed at the 1~10 µg doses (**Figure 8e**), and no statistical differences in CD8 Tcm were observed between doses (**Figure 8c**). Also, an increase in the percentage of CD8 effector memory T cells (Tem) was observed at the 5 and 10 µg doses (**Figure 8b**). STP2104 immunization significantly increased the percentage of germinal center (GC) B cells at the 10 µg dose, producing memory B cells and long-lived plasma cells, which produce antigen-specific antibodies, including neutralizing antibodies (**Figure 8f**).

In summary, STP2104 was highly immunogenic in mice, as strongly antigen-binding IgG1 and IgG2a were observed 4 weeks after priming as well as 3 weeks after boosting, and a higher NAb titer was strongly elicited. Moreover, potent NAb responses were observed together with a Th1-phenotype CD4^+^ response, as well as IFN-γ and CD8^+^ T-cell responses, after immunization.

### 3.8 The STP2104 vaccine protected hACE2 Mice from a SARS-CoV-2 lethal challenge

**Figure 9:**
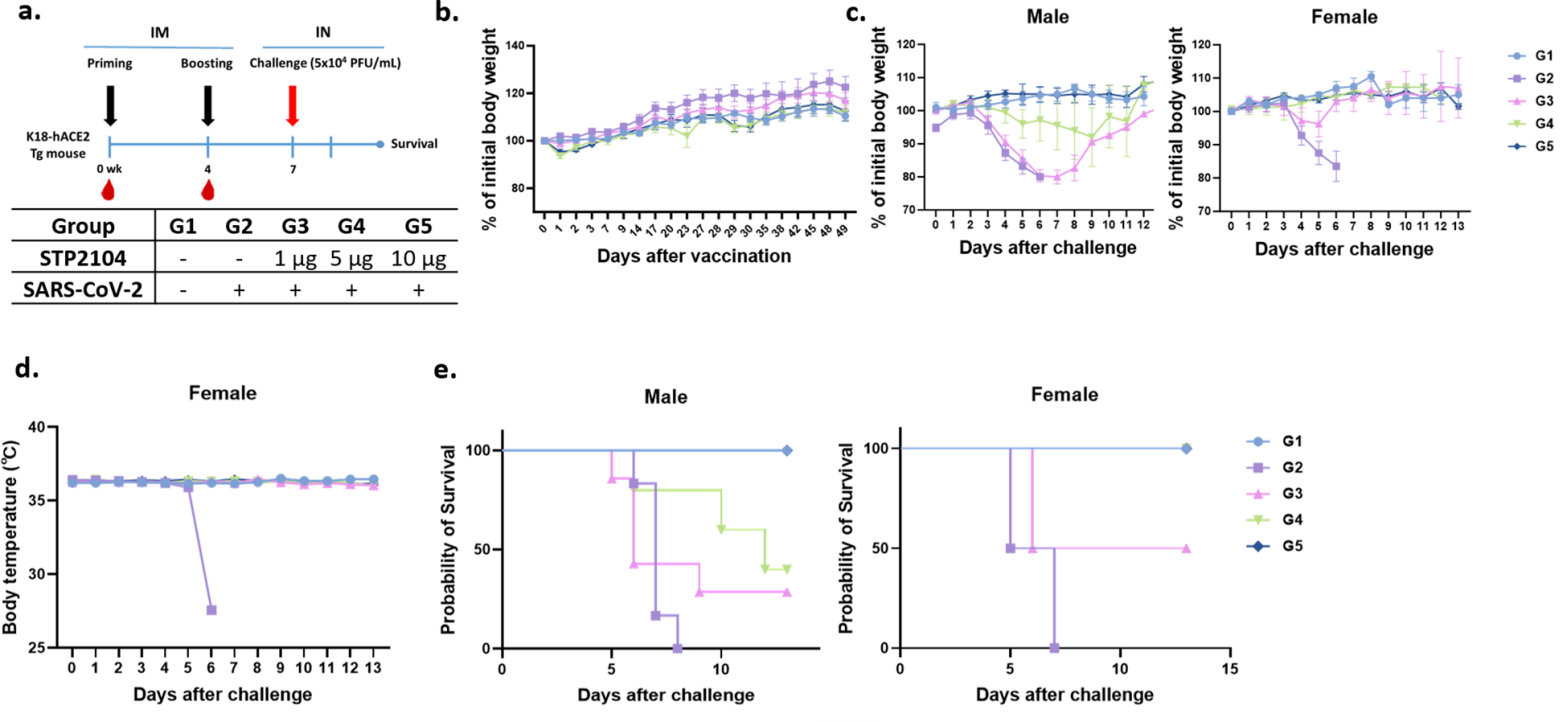
Protection efficacy of STP2104 against SARS-CoV-2 lethal challenge in K18-hACE2 transgenic mice. **(a)** Overall study scheme. Female K18-hACE2 transgenic mice were intramuscularly immunized with 1 μg, 5 μg, or 10 μg at 0 and 4 weeks and then intranasally challenged with 5×10^4^ PFU/mL of SARS-CoV-2 at 7 weeks after the first immunization. (n = 10/group). **(b)** All mice (n = 9–10/group) were monitored twice a week for body weight and any symptoms. After the beginning of immunization, the body weight and body temperature of mice were measured until recovery. **(c–e)** The K18-hACE2 male (left, n = 6) and female (right, n = 3–4) mice’s **(c)** body weights, **(d)** temperature changes (in the female group), and **(e)** mortality were monitored daily for up to 13 days post-challenge. For the K18-hACE2 female mice only, an at least 5 μg dose of STP2104 provided 100% protection against lethal challenge.

Based on the significantly higher levels of humoral and cell-mediated immune responses in the mice after immunization, we further investigated whether STP2104 could protect human ACE2 transgenic mice from a lethal challenge of the SARS-CoV-2 virus via the intranasal (I.N.) route. Mice were divided into five groups and immunized as depicted in **Figure 9a**. In Groups 4 and 5, a slight reduction in body weight was observed the day after the second vaccination, but the mice fully recovered this weight within 1~2 days (**Figure 9b**). Three weeks after boosting, all the mice in Groups 2–5 were I.N. challenged with SARS-CoV-2 (type S) at 5 × 10^4^ PFU per mouse (20 μL). Both male and female mice in the non-vaccinated, challenged group, Group 2, displayed a significant reduction in body weight and experienced hypothermia, resulting in 100% lethality by 8 days post-challenge (**Figures 9c–e**). When the mice were immunized with 10 μg of STP2104, no clinical symptoms were observed, and 100% of the mice survived the SARS-CoV-2 challenge. Interestingly, we observed a substantial difference in protective efficacy between the male and female mice immunized with 5 μg of STP2104 (Group 4). While the female mice survived at a rate of 100%, the survival rate of male mice was only 50%. A similar trend was observed in Group 3 (1 μg of STP2104); the survival rates were 50% and 33% for female and male mice, respectively. These results indicate that sex-based immunological differences result in susceptibility to SARS-CoV-2 and the response to vaccines.

### 3.9 STP2104 vaccine significantly reduces viral burden

**Figure 10:**
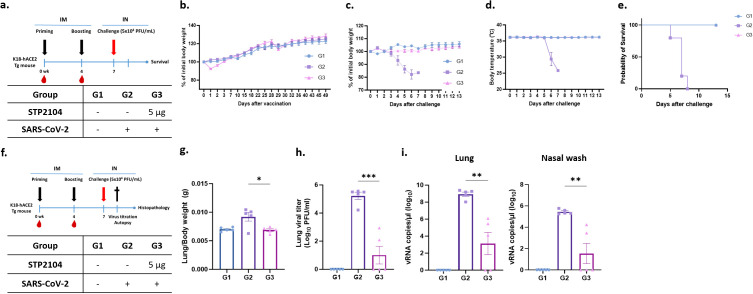
Residual viral titer after STP2104 vaccination followed by SARS-CoV-2 lethal challenge in hACE2 mice. **(a)** Schedule for STP2104 immunization and viral challenge in K18-hACE2 residual viral titers after STP2104 vaccination followed by SARS-CoV-2 lethal challenge in hACE2 mice. **(a)** Schedule for STP2104 immunization and viral challenge for K18-hACE2 mice. **(b)** The change in body weight after vaccination (n = 10/group). All mice were monitored twice a week for bodyweight and any symptoms. After the beginning of immunization, the mice’s bodyweights were measured until the weight had been regained. After the K18-hACE2 female mice were challenged (n = 5/group), **(c)** body weight, **(d)** body temperature changes, and **(e)** survival rate was monitored daily for up to 13 days post-challenge. **(f)** To assess residual viral burden after immunization followed by a challenge in the K18-hACE2 female mice, lung tissues and nasal wash discharge were harvested at 4 days p.c. The K18-hACE2 female mice’s (n = 5/group) bodyweight and body temperature changes were monitored daily for up to 4 days p.c. (data not shown). **(g)** Lung-to-body weight ratio was measured at 4 days p.c. **(h)** Infectious residual SARS-CoV-2 titer in lung tissues was confirmed by means of a plaque assay. **(i)** Viral RNA was amplified via qRT-PCR using the total RNA extracted from right lung tissues (left) and nasal wash discharge (right). The viral RNA copy numbers were calculated using the SARS-CoV-2 RNA standard sample. The results were statistically analyzed using a *t*-test (*p < 0.05, **p < 0.01, and ***p < 0.001).

Based on the above findings, we further confirmed the protective efficacy of STP2104 immunization, checked for the presence of residual virus on lung and nasal swabs, and performed a pathologic examination with a 5 μg dosage within the same experimental scheme (**Figure 10a**). As expected, the vaccinated group displayed decreased body weight after immunization and recovered quickly (**Figure 10b**). After a lethal-dose challenge with SARS-CoV-2, the mice in Group 2 showed significant clinical symptoms and died in 100% of cases, while immunized mice in Group 3 survived at a rate of 100% without any weight loss or changes in body temperature (**Figures 10c–e**).

To assess viral burden and histopathology, the mice were sacrificed at 4 days p.c., and samples were collected (**Figure 10f**). As shown in previous experiments, mice immunized with 5 μg of STP2104 did not show any changes in body weight after a viral challenge (**Figures 10b, c**); this was especially true for females (**Figure 9c**). The ratios of lung weight to body weight for the mice significantly increased in Group 2, indicating pulmonary edema (**Figure 10g**). However, the ratio for the vaccinated group, Group 3, was comparable to that for the uninfected mice in Group 1. We quantified the infectious viral load in homogenized lung tissues using a plaque assay and found that there was a significant reduction in the lung viral titer in Group 3 compared to that in Group 2. In addition, the viral titers of three out of the five mice in Group 3 were under the limit of detection, which was as low as 25 PFU/mL. These results were further elucidated via qRT-PCR of viral nucleocapsid (N) genes in lung tissues and through nasal washes. Consistent with the lung viral titer results, remarkably lower levels of viral RNA were detected in the lung tissues and nasal wash discharge of Group 3 (**Figure 10i**). These results suggest that the immunization of STP2104 (5 μg) twice in four-week intervals induces protective immunity and is sufficient to provide complete protection against SARS-CoV-2 lethal infection (**Figure 10e**).

After STP2104 administration, temporary weight loss was observed; the mice regained this weight within 1–2 days (**Figure 10b**). From days 4 to 5 after the SARS-CoV-2 challenge, the body weight (day 4) and body temperature (day 6) in Group 2 (vehicle) rapidly decreased. However, there was neither a decrease in body temperature nor any weight loss in Group 3 (STP2104 5 μg) (**Figures 10c, d**). As a result of confirming the survival rate after SARS-CoV-2 infection, the STP2104-administered Group 3 mice (STP2104 5 μg) showed a 100% survival rate, while the Group 2 mice (vehicle) died on day 8 (**Figure 10e**). Pulmonary edema induced by SARS-CoV-2 infection was statistically significantly reduced in the Group 3 mice (STP2104 5 μg) inoculated with STP2104, showing a similar lung-to-body weight ratio to those in Group 1 (non-challenged) (**Figure 10g**). On day 4 post-inoculation, significantly less residual virus was observed on lung and nasal swabs for Group 3 (STP2104 5 μg) compared to that for Group 2 (vehicle) (**Figures 10h, i**).

### 3.10 Pulmonary lesions caused by SARS-CoV-2 were prevented by STP2104

**Figure 11:**
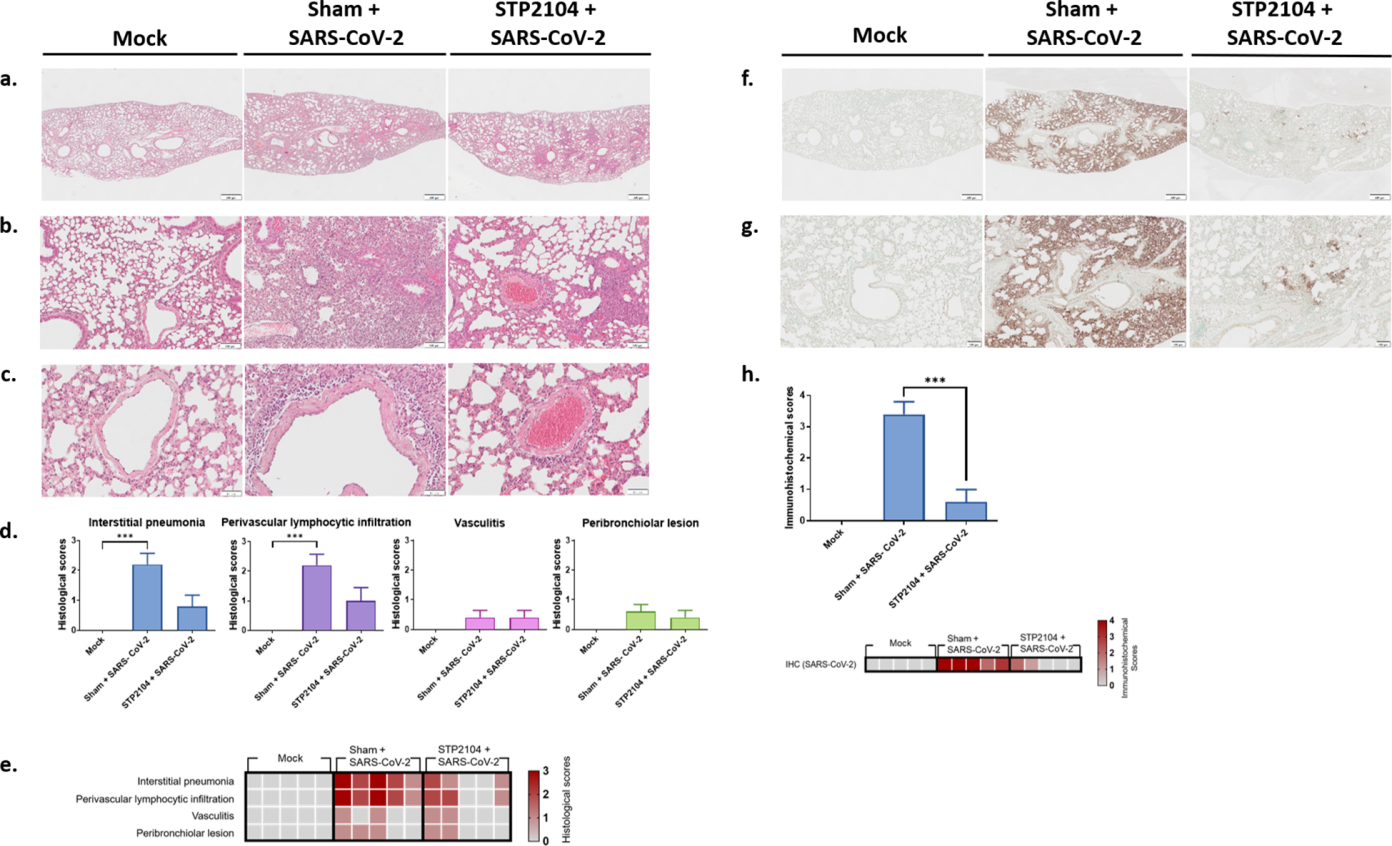
Histological and immunohistochemical features of the lungs from human ACE2 transgenic mice. **(a)** Lower and **(b)** higher magnifications of microscopic observations of lung parenchyma from the mock or sham + SARS-CoV-2 and STP2104 + SARS-CoV-2 groups. Interstitial pneumonia, perivascular lymphocytic infiltration, AND **(c)** vasculitis were evaluated. Scale bars: 500 μm **(a)**, 100 μm **(b)**, and 50 μm **(c)**. **(d)** Histologic scoring of the lung. Data are presented as means ± SE. *** p < 0.001. **(e)** Heatmap representing the histological scores of various pulmonary changes. **(f)** Lower and **(g)** higher magnifications of the immunohistochemistry of lung tissue. Scale bars: 500 μm **(f)** and 100 μm **(g)**. **(h)** IHC scoring and heatmap of the lungs. Percentages of the stained area were scored. Data are presented as means ± SE and statistically analyzed using a *t*-test (***p < 0.001).

Histopathologic examination revealed that the pulmonary histology of the mock group was normal. However, in the sham + SARS-CoV-2 group, interstitial pneumonia was severe (**Figures 11a, b, d**). There was diffuse infiltration of macrophages, lymphocytes, plasma cells, and occasional eosinophils in the alveolar space, and the alveolar walls were thickened by inflammatory infiltrates. In the peribronchiolar region, mild lymphocytic infiltration was observed, with occasional sloughed epithelial cells and macrophages in the bronchiolar lumen (**Figures 11b, d**). Moreover, the perivascular space was loosely distended with lymphocytic infiltration (**Figures 11c, d**). There were some activated endothelial cells and adhered lymphocytes on the endothelial surface, with occasional necrotic cells and lymphocytes in the vascular lumen (**Figures 11c, d**). Compared to the sham + SARS-CoV-2 group, the severity of interstitial pneumonia and the perivascular lesion was much lower in the STP2104 + SARS-CoV-2 group (**Figure 11d**, **Supplementary Tables S1**, **S2**). These pulmonary lesions were scored according to microscopic scoring criteria (**Figure 11e**, **Supplementary Table S1**). The sham + SARS-CoV-2 group showed significantly more severe interstitial pneumonia and perivascular lymphocytic infiltration compared to the mock group (*p* < 0.001). Furthermore, the STP2104 + SARS-CoV-2 group showed no significant differences in interstitial pneumonia and perivascular lymphocytic infiltration compared to the mock group, indicating the protective effect of STP2104 in SARS-CoV-2-induced pulmonary lesions. Immunohistochemistry (IHC) revealed that, in contrast to the mock group, a strong IHC-positive reaction against the SARS-CoV-2 N protein was present on the alveolar epithelium and luminal inflammatory cells in the sham + SARS-CoV-2 group (**Figures 11f, g**). However, only a small number of positive areas on the alveolar epithelium were detected in the STP2104 + SARS-CoV-2 group. In **Figure 11h**, the IHC scoring results show a significantly smaller proportion of the IHC-positive area in the STP2104 + SARS-CoV-2 group compared to that in the sham + SARS-CoV-2 group (*p* < 0.001).

## 4 Discussion

In this paper, we developed a COVID-19 mRNA vaccine, STP2104, using SmartCap^®^ SC101 selected through the capping library screening (CLS) method. The *in vitro* potency, *in vivo* immunogenicity, and protection efficacy of the STP2104 mRNA vaccine platform developed using the novel 5′-cap analogue SC101 were verified in mice and rats. Various reporter mRNAs (e.g., eGFP, hEPO, and fLUC) were co-transcriptionally synthesized using various SmartCap^®^s and evaluated in a comparison with CleanCap^®^ (CC) reagent AG. The synthesized mRNAs were analyzed for their mRNA yields, 5′-capping efficiency, and potency. Based on the protein expression efficiency determination in HEK293T, Huh7, RD, or C2C12 cells, SC101 was selected as the 5′-capping reagent for use in the further development of the COVID-19 mRNA vaccine STP2104.

We observed that the specific 5′-cap analogue-driven reporter protein expression efficiency differed depending on the coding sequence (CDS) and cell line used for analysis. Several other similar observations and reasons for the differential potency have been previously reported. First, the methylation of the first transcribed nucleotide significantly affects mRNA expression in dendritic cells but not in HeLa or 3T3-L1 cells (18). The eIF4F complex, which consists of eIF4E, eIF4G, and eIF4A, initiates translation by binding to the cap structure, and the affinity of these proteins for the methylated cap varies (17). Additionally, the expression level of these genes differs among cell lines. Second, the influence of the cap motif on the decapping enzyme hDcP2 varies (16, 17). Furthermore, the expression level of the hDcP2 gene differs among different cell types, leading to differences in CDS expression. Third, impurities in *in vitro*-transcribed (IVT) products, such as double-stranded RNA (dsRNA) and uncapped mRNA, significantly affect protein expression. Differences in the expression of the IFIT1 gene, reflecting these impurities, can result in varying expression across cell lines (18). Fourth, differences in the expression of the CAPAM methyltransferase gene can lead to variations in cap-analogue-mediated CDS expression (16, 17). In summary, these differences can be attributed to the unique cellular environments that regulate gene expression, including the expression of specific transcriptional and RNA-binding proteins, cell cycle stages, and RNA metabolic pathways. In addition, certain cells may exhibit immune responses to cap analogues, affecting RNA expression levels, while other immune regulatory factors specific to the cell type may influence RNA stability and translation efficiency, leading to expression differences. Therefore, the SmartCap^®^ library screening approach is an appropriate process for the development of mRNA vaccines and therapeutics, allowing one to select the best-performing 5′-cap analogue, which is dependent on the gene of interest and the cells, tissues, or organs targeted. Although our SmartCap^®^ SC101 is a synthetic cap with one 2′-fluoro substituted at the second ribose of adenosine (**Figure 2b**), the potency of SC101 was at least comparable to that of CC under ST Pharm’s optimized IVT reaction conditions.

To determine the immunogenicity of STP2104, BABL/c mice (female) were I.M. inoculated with STP2104 at 1 μg, 5 μg, or 10 μg doses twice at 3-week intervals. STP2104 is highly immunogenic in mice, as balanced, strongly antigen-binding IgG1 and IgG2a were observed 4 weeks after priming as well as 3 weeks after boosting. Moreover, potent NAb responses were observed together with a Th1-phenotype CD4^+^ response, as well as IFN-γ and CD8^+^ T-cell responses, after immunization. STP2104 immunization significantly increased the proportion of germinal center (GC) B cells, memory B cells, and long-lived plasma cells, which provoked various B cells to produce antigen-specific antibodies, including NAbs. Based on its ability to produce effective NAbs, STP2104 demonstrated excellence in promoting humoral and cellular immunity by confirming the IFN-γ of CD8 effector T cells as well as the Th1-phenotype CD4^+^ helper T cells.

To evaluate cell-mediated immunity, we synthesized five peptide pools (PPs) of the spike protein and confirmed cellular immune response via the ELISpot assay with PP2 including a receptor binding domain (RBD) of the spike protein, which is a strong stimulant for cell-mediated immunity. However, unlike the humoral immune response, the highest activity of IFN-γ-producing T cells was observed at a dose of 5 μg instead of 10 μg. Furthermore, the highest ratio of CD8^+^ Tem was observed at a dose of 5.0 μg, not 10 μg. Although there have been no reports on normal animal prophylactic mRNA vaccines, T-cell responses against excessive antigen doses have been reported. Consistent with our findings, previous studies have shown that high antigen doses can attenuate vaccine-specific T-cell responses. The administration of a high dose of HIV antigens, in combination with cationic liposomal adjuvants, results in a reduction in the quantity of polyfunctional T cells producing IFN-γ and TNF-α compared to that available following lower doses. In a mouse model of tuberculosis, high antigen doses negatively affected the efficacy of post-exposure vaccines against tuberculosis infection, a phenomenon attributed to the terminal differentiation and decreased functional avidity of T cells (28). Moreover, there are some reports indicating that high-dose immunization can lead to immune tolerance phenomena, such as T-cell anergy (29–31). In this case, it is known that the antigen reactivity of T cells tends to decrease. As another piece of supporting evidence, T-cell exhaustion was observed in SARS-CoV-2-infected patients (32, 33), including after mRNA vaccination with an additional booster (34). Several studies have reported that repeated immunizations and breakthrough infections (BTIs) boosted Ab responses, but T-cell responses were not enhanced by frequent dosing (34, 35), even in cancer patients (36). Therefore, precise modulation of vaccine dosing is essential to optimize T-cell responses, as excessive antigen doses can lead to clonal deletion, immune tolerance, terminal differentiation, or the exhaustion of T cells (37).

In addition, we evaluated the protection efficacy of human ACE2 Tg mice against a SARS-CoV-2 viral challenge after a 1, 5, or 10 μg dose of the STP2104 mRNA vaccine. The vaccination enhanced the survival rate dose-dependently, lowered the residual viral titers in lung tissues and nasal wash discharge, and reduced lung histopathological scores. Elsewhere, the efficacy and safety of injecting 0.2, 1, and 5 μg of BNT162b2 in mice and 30 and 100 μg of BNT162b2 in rhesus macaques were confirmed. In addition, vaccination with 0.01, 0.1, or 1 μg of mRNA-1273 in a mouse challenge model confirmed its immunogenicity and safety (38). Following immunization with 0.25, 1, 2, or 4 μg of CVnCoV, it was found that that a 2 or 10 μg dose in golden hamsters led to dose-dependent protection efficacy. Moreover, there were no signs of vaccine-associated enhanced disease (VAED) in the hamster challenge model (39). Based on various non-clinical study results, STP2104 has been proven to be highly immunogenic and protective against SARS-CoV2 viral challenge.

Furthermore, the pre-clinical toxicity of STP2104, which is co-transcriptionally synthesized with SmartCap^®^ SC101, in animals and its safety, tolerability, and immunogenicity in humans need to be assessed in order to verify that it is less toxic, safe, and potent in humans. Since COVID-19 is no longer considered a pandemic and the currently prevalent variant is still undergoing mutations, our STP2104 mRNA vaccine needs to be developed as a booster vaccine with the most updated variant S sequence. In this report, we introduce a novel SmartCap^®^ library screening method, a powerful way of selecting the best-performing 5′-cap analogue for the development of mRNA vaccines/therapeutics.

## Data Availability

The raw data supporting the conclusions of this article will be made available by the authors, without undue reservation.

## Supplementary material

Supplementary Table 1 Microscopic scoring criteria for pulmonary lesions (related to **Figures 11D, E**).

Supplementary Table 2 Individual scoring of histopathologic readings based on the scoring criteria (related to **Figures 11D, E** and **Supplementary Table S1**).
