# Supplementary material for: Discovery and development of a safe and efficient COVID-19 mRNA vaccine, STP2104, using a novel capping library screening method
Source: Front Immunol. 2025 Jun 9;16:1571713. doi: 10.3389/fimmu.2025.1571713 (PMC12183165; doi:10.3389/fimmu.2025.1571713)
Supplement: Supplementary Table 1 — Microscopic scoring criteria for pulmonary lesions (related to Figures 11D, E ). [file Table1.docx]

Supplementary Material

# Supplementary Tables

**Supplementary Table 1:** Microscopic scoring criteria for pulmonary lesions (related to Figure 11d,e).

| **Lesion** | **Description** |
| --- | --- |
| **Interstitial pneumonia** | There is diffuse infiltration by macrophages, lymphocytes, plasma cells, and, occasionally, eosinophils in the alveolar space, and alveolar walls are thickened by inflammatory infiltrates. |
| **Perivascular edema with lymphocytic infiltration** | The perivascular space is loosely distended, and lymphocytic infiltration is evident. |
| **Vasculitis** | The mononuclear inflammatory cells are present in the vascular lumen and endothelial surface. |
| **Peribronchiolar lymphocytic infiltration** | There are sporadically present sloughed epithelial cells in the bronchiolar lumen, and mild peribronchiolar lymphocytic infiltration is evident. |

**Supplementary Table 2.** Individual scoring of histopathologic readings based on the scoring criteria (related to Figure 11d,e and Supplementary Table 1).

|  | | **Interstitial pneumonia** | **Perivascular edema with lymphocytic infiltration** | **Vasculitis** | **Peribronchiolar lymphocytic infiltration** |
| --- | --- | --- | --- | --- | --- |
| **G1** | #1 | 0 | 0 | 0 | 0 |
|  | #2 | 0 | 0 | 0 | 0 |
|  | #3 | 0 | 0 | 0 | 0 |
|  | #4 | 0 | 0 | 0 | 0 |
|  | #5 | 0 | 0 | 0 | 0 |
|  | **Average** | **0** | **0** | **0** | **0** |
| **G2** | #1 | 3 | 3 | 1 | 1 |
|  | #2 | 2 | 2 | 0 | 1 |
|  | #3 | 3 | 3 | 1 | 1 |
|  | #4 | 2 | 2 | 0 | 0 |
|  | #5 | 1 | 1 | 0 | 0 |
|  | **Average** | **2.2** | **2.2** | **0.4** | **0.6** |
| **G3** | #1 | 2 | 2 | 1 | 1 |
|  | #2 | 1 | 2 | 1 | 1 |
|  | #3 | 0 | 0 | 0 | 0 |
|  | #4 | 0 | 0 | 0 | 0 |
|  | #5 | 1 | 1 | 0 | 0 |
|  | **Average** | **0.8** | **1** | **0.4** | **0.4** |
| (0: normal, 1: mild, 2: moderate, 3: severe)  **Grade 1 (Minimal):** This grade corresponds to a histopathologic change that ranges from inconspicuous to barely noticeable but is so minor, small, or infrequent as to warrant no more than the lowest assignable grade.  For multifocal or diffusely distributed lesions, this grade was used for processes where less than approximately 10% of the tissue in an average high-power field was involved.  **Grade 2 (Mild):** This grade corresponds to a histopathologic change that is a noticeable but not prominent feature of the tissue.  For multifocal or diffusely distributed lesions, this grade was used for processes where between approximately 10% and 25% of the tissue in an average high-power field was involved.  **Grade 3 (Moderate):** This grade corresponds to a histopathologic change that is a prominent but not dominant feature of the tissue.  For multifocal or diffusely distributed lesions, this grade was used for processes where between approximately 25% and 50% of the tissue in an average high-power field was involved. | | | | | |
